# Red Alga *Porphyridium* Supports High‐Yield Production of a Functional Chimeric Hepatitis B Surface Antigen With Strong Cellular and Humoral Immunogenicity

**DOI:** 10.1111/pbi.70270

**Published:** 2025-07-22

**Authors:** Ana‐Maria Pantazica, Alexander Hammel, Iuliana Caras, Irina Ionescu, Catalin Tucureanu, Adrian Onu, Maria Murace, Jihong Liu Clarke, Crina Stavaru, Norica Branza‐Nichita, Ralph Bock

**Affiliations:** ^1^ Institute of Biochemistry of the Romanian Academy Bucharest Romania; ^2^ Max‐Planck‐Institut für Molekulare Pflanzenphysiologie (MPI‐MP) Potsdam‐Golm Germany; ^3^ “Cantacuzino” Medico‐Military National Research Institute Bucharest Romania; ^4^ Max‐Planck‐Institut für Kolloid‐ Und Grenzflächenforschung (MPI‐ KG) Potsdam‐Golm Germany; ^5^ NIBIO–Norwegian Institute of Bioeconomy Research Ås Norway

**Keywords:** chimeric antigen, HBV, hepatitis B, immunity, microalga, molecular farming, *Porphyridium purpureum*, Rhodophyta, vaccine

## Abstract

Microalgae represent promising production factories for the light‐driven, cost‐effective production of recombinant proteins. The red microalga *Porphyridium purpureum* displays particularly favourable transgene expression properties due to the episomal maintenance of transformation vectors at high copy numbers in the nucleus. In this work, we explored the potential of *Porphyridium purpureum* to synthesise a candidate vaccine against Hepatitis B virus (HBV). We show high‐yield expression of an HBV chimeric surface antigen and efficient assembly of virus‐like particles (VLPs) in algal cells. We established a purification protocol for the VLPs and conducted vaccination studies in experimental animals. The results demonstrate that the alga‐produced HBV antigen elicits superior humoral and cellular immune responses compared to a commercial HBV vaccine produced in yeast. The antigen triggers virus‐neutralising antibodies against different HBV variants, including vaccine‐escape mutations that evade the immune response to current vaccines in humans. Our work establishes *Porphyridium* as a highly promising production platform for vaccines and other proteinaceous biopharmaceuticals.

## 1 Introduction

Microalgae have emerged as promising platforms to produce recombinant proteins, including enzymes and proteinaceous biopharmaceuticals. They offer the advantage of photoautotrophic growth, can be produced by employing bioreactor‐based microbial cultivation techniques, and possess the ability to perform complex eukaryotic‐type post‐translational modifications. In addition, the cultivation of microalgae synthesising recombinant therapeutics poses minimal safety concerns and is scalable at low costs (Banerjee and Ward 2022; Barbosa et al. 2023). Despite these advantages, progress in microalgal biotechnology has been limited by the lack of molecular tools for high‐level expression of recombinant proteins in suitable host species. Owing to its ease of cultivation and genetic manipulation, *Chlamydomonas reinhardtii* is currently the most widely used microalga for heterologous protein expression. However, the low recombinant protein yields achieved in this system remain a major drawback (Scaife et al. 2015).

Recently, studies in the red microalga *Porphyridium purpureum* have shown great promise for recombinant protein production. The alga maintains transformed plasmids episomally at high copy numbers, resulting in high transgene expression levels, with protein yields reaching up to 5% of the total soluble protein (TSP) (Li and Bock 2018; Hammel, Neupert, and Bock 2024). Moreover, successful expression of complex proteins, such as the Hepatitis C Virus (HCV) envelope glycoprotein E2, has been achieved in *P. purpureum* (Hammel, Cucos, et al. 2024). The alga‐derived HCV‐E2 was demonstrated to be properly folded, undergo decoration with mammalian cell‐like N‐linked glycans, and act as a strong immunogen in experimental animals. Together, these findings have suggested *P. purpureum* as a competitive new production system for the biosynthesis of proteins for biomedical applications (Hammel, Cucos, et al. 2024).

In this work, we have investigated the capacity of *P. purpureum* to support the production of more complex viral antigens that assemble into high‐molecular weight virus‐like particles (VLPs), to be employed as potential subunit vaccine candidates. As a test system, we used a recently developed Hepatitis B Virus (HBV) chimeric surface antigen with strong immunogenic properties when produced in either mammalian cells or transiently expressed in *Nicotiana benthamiana* leaves (Pantazica et al. 2022, 2023). The development of improved vaccines remains a high priority in the worldwide efforts to combat HBV infections, which cause severe liver disease and over 800 000 deaths every year. Conventional vaccines, based on the HBV small (S) envelope protein produced in yeast, are efficient in about 90% of healthy adult recipients. However, < 75% of patients with comorbid diseases develop protective anti‐HBV antibody titers (Vesikari et al. 2023). Additionally, there are other limitations, including low‐level long‐term protection and the appearance of vaccine‐escape mutations (VEMs) that evade the immune response, that need to be urgently addressed (Bian et al. 2013; Lai et al. 2012). Efforts to tackle this emergency have eventually led to the approval of the first three‐antigen HBV vaccine that, in addition to S, includes the other two envelope proteins, the middle (M) and the large (L) protein, both of which display highly immunogenic epitopes. This vaccine was shown to induce protective antibody levels also in non‐responders to the S‐only vaccine (Vesikari, Finn, et al. 2021; Vesikari, Langley, et al. 2021).

The HBV envelope proteins are able to form highly immunogenic subviral envelope particles (SVPs), collectively known as the HBsAg, both in infected patients and upon synthesis in recombinant systems in the absence of other viral factors (Patient et al. 2009). We have recently shown that this property is retained by chimeric L/S antigens that are based on the insertion of relevant immunogenic epitopes derived from the preS1 domain of the L protein into the antigenic loop of S (Pantazica et al. 2022). One of these antigens, the HBV‐S/preS1^16‐42^ chimera, has triggered a significant immune response in vivo and induced antibodies with high neutralising activity against both wild‐type (WT) and VEM HBV variants, validating this approach as a new strategy for the development of improved HBV vaccine candidates (Pantazica et al. 2022, 2023). However, the production platforms explored so far are either very costly (mammalian cells) or unable to achieve high yields of the chimeric HBV antigens (transient expression in plants), despite the strong immunogenic properties of the synthesised antigens (Dobrica et al. 2017; Pantazica et al. 2022, 2023).

Here, we show that the red alga *P. purpureum* supports high expression levels of the HBV‐S/preS1^16‐42^ antigen. The chimeric HBV antigen is assembled into VLPs, which are specifically recognised by conformational anti‐S antibodies, indicating appropriate protein folding in algal cells. Moreover, we demonstrate that the alga‐produced HBV antigen elicits superior humoral and cellular immune responses in mice as compared to a commercial S protein‐based HBV vaccine produced in yeast and triggers virus‐neutralising antibodies against both WT and VEM HBV variants.

## 2 Results

### 2.1 HBV Antigen Design and Expression in *P. purpureum*

In mammalian cells, the HBV‐S protein is co‐translationally inserted into the endoplasmic reticulum (ER) membrane by the uncleaved N‐terminal targeting sequence and translocation/anchor signals located within the first and second transmembrane domains (TMD), respectively (Prange 2022; Seitz et al. 2020). Shortly after synthesis, the S protein forms disulfide‐bridged dimers that serve as building blocks for SVP assembly at the ER–Golgi Intermediate Compartment (ERGIC), followed by secretion via the constitutive secretory pathway (Huovila et al. 1992).

**FIGURE 1:**
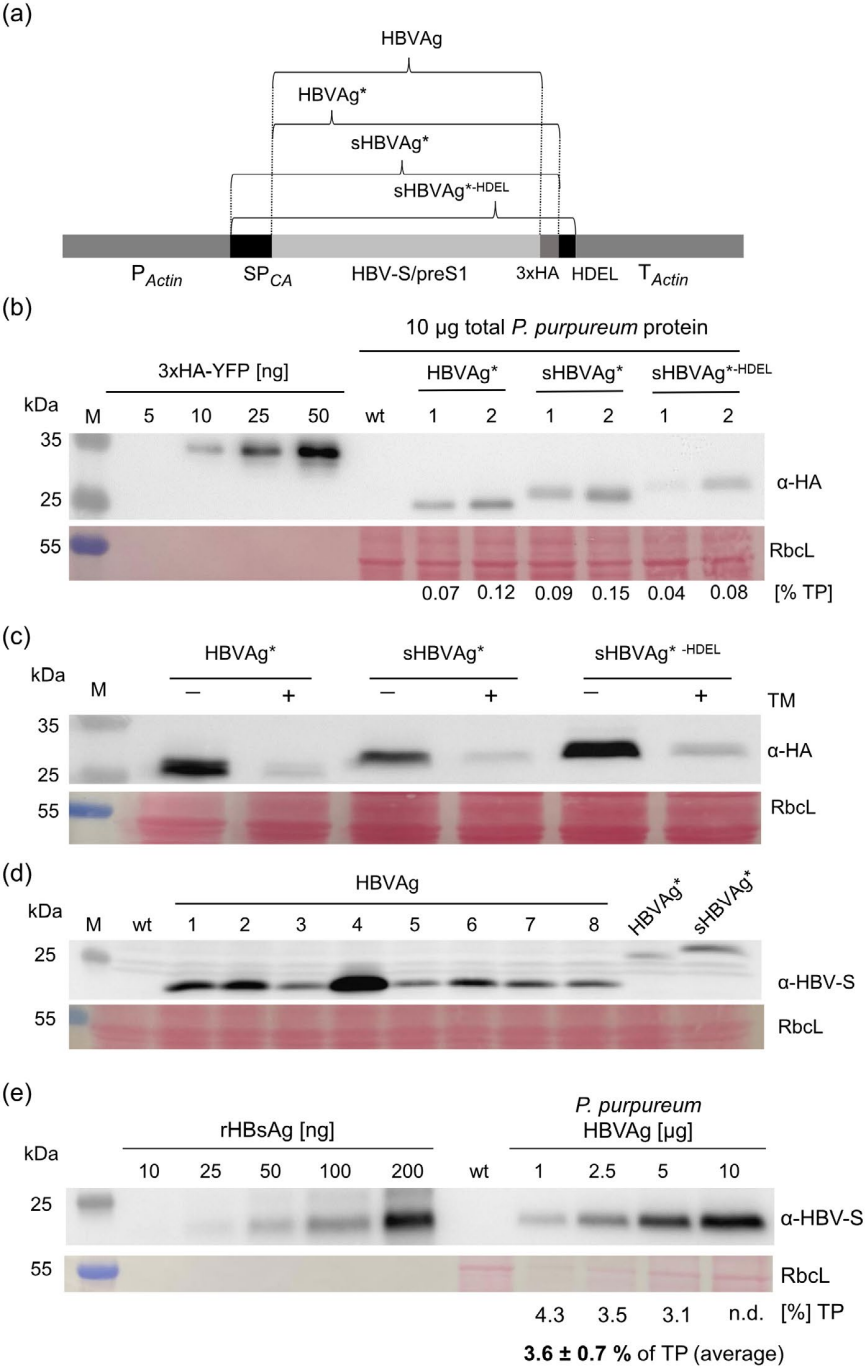
Physical map, expression and glycosylation of HBV‐S/preS1 antigen variants in *P. purpureum*. (a) Physical map of the expression cassette. The HBV‐S/preS1 sequence was codon optimised for the nuclear genome of *P. purpureum* and expressed under the control of the native *Actin* promoter (P_*Actin*_) and terminator (T_*Actin*_). In addition to the untagged (HBVAg) version, the antigen was produced as a 3xHA‐tagged version (HBVAg*) to facilitate early‐stage expression screening. Additionally, ER‐targeted (sHBVAg*) and ER‐retained (sHBVAg*^‐HDEL^) 3xHA‐tagged variants were constructed by incorporating the endogenous signal peptide of carbonic anhydrase 1 (SP_CA_) and addition of a C‐terminal HDEL motif. (b) Immunoblot analysis of the HBVAg* variants expressed in *P. purpureum* . Samples of 10 μg total cellular protein of two independent transgenic lines per antigen variant were analysed by immunoblotting using an anti‐HA antibody (α‐HA) after protein separation by 12% SDS‐PAGE. The region of the Ponceau‐stained membrane contains the large subunit of Rubisco (RbcL), which served as loading control. A dilution series of recombinant 3xHA‐tagged yellow fluorescent protein (YFP) was used as standard for semi‐quantitative assessment of expression levels. Estimated accumulation levels of the antigen are shown below each lane (in percent of the total protein; TP). M: Molecular weight marker. (c) Cells expressing the different HBVAg* variants were grown in medium with 1 μg/mL tunicamycin (TM) (+) or without TM (−) for 16 h. Samples of 50 μg total protein were analysed by immunoblotting with an anti‐HA antibody after protein separation by 12% SDS‐PAGE. Equal loading was verified by Ponceau staining of the membrane, showing the RbcL region. M: Molecular weight marker. (d) Immunoblot analysis of independent transgenic strains expressing the untagged HBVAg. Samples of 20 μg total protein were loaded on the gel along with the 3xHA‐tagged variants (HBVAg* and sHBVAg*) as controls and detected using an anti‐HBV S antibody. The Ponceau staining shows the RbcL‐containing region of the membrane. M: Molecular weight marker. (e) Antigen quantification using a recombinant HBV‐S protein as standard. Different amounts of a recombinant HBV‐S protein standard were loaded next to the indicated amounts of protein samples from cells expressing the untagged HBVAg and analysed by immunoblotting using an anti‐HBV S antibody. The numbers below the blot indicate the calculated yield in % of the total protein (TP). The average yield was 3.6% of TP. The Ponceau staining of the membrane shows the region containing RbcL. M, molecular weight marker; n.d., not determined; wt, wild type.

To assess the molecular determinants for optimal expression of the HBV‐S/preS1^16‐42^ antigen in *P. purpureum* (hereby referred to as HBVAg), transformation vectors were constructed to target the antigen to different subcellular destinations. To facilitate antigen detection, all constructs contained a C‐terminally located 3xHA tag (HBVAg*; Figure 1a). In all cases, transcription was under the control of the actin promoter and terminator sequences from *P. purpureum* . The HBVAg* construct contains the sequence encoding the HBV‐S/preS1^16‐42^ antigen and should be targeted to the ER, if the TMD‐located translocation signals are functional in red alga cells. Two additional variants were designed to (i) facilitate ER translocation by adding the endogenous carbonic anhydrase 1 (CA1) signal peptide to the N‐terminus of the antigen (sHBVAg*) and (ii) confer ER retention by incorporating an additional C‐terminal HDEL‐signal (sHBVAg*^‐HDEL^; Figure 1a). Moreover, as insertion of C‐terminal tags was previously shown to hinder HBV assembly (Pastor et al. 2019), we also constructed a tag‐free version of the antigen to be used for molecular characterisation and in later immunisation studies (HBVAg, Figure 1a).

Algal transformation experiments were conducted using particle gun‐mediated (biolistic) DNA delivery. We first analysed the HA‐tagged HBV antigens to assess the subcellular localisation of the antigen and the functionality of the ER translocation signal. For each construct, several transgenic algal clones were isolated. HBV antigen accumulation levels were determined by semiquantitative immunoblotting using 3xHA‐tagged YFP as a standard (Figure 1b). Antigen accumulation was detected for all variants transformed into *P. purpureum* , at levels reaching up to 0.15% of the total protein (TP). The variation observed in the two lines expressing the sHBVAg*^‐HDEL^ antigen is likely explained by the presence of residual wild‐type cells in the primary transformed algal colonies, which is commonly observed in *Porphyridium* transformation (Hammel, Cucos, et al. 2024; Hammel, Neupert, and Bock 2024).

The HBV‐S protein contains a conserved N‐glycosylation site at Asn146, which is modified in approximately half of the molecules present in infected patients and transfected mammalian cells, and to a lesser extent when expressed in plants (Dobrica et al. 2020). By contrast, the S protein remains unglycosylated in yeast, the main biotechnological platform for the production of commercial HBV vaccines (Cregg et al. 1987). Interestingly, a slight variation of the apparent molecular weight was observed between the HBVAg* constructs, which could result from the attachment of an N‐linked oligosaccharide to the conserved site in the ER, or alternatively, from the unsuccessful cleavage of the CA1‐derived signal peptide from the HBV antigen (Figure 1b). To distinguish between these possibilities, algal cells were grown in the presence of tunicamycin (TM), a potent protein N‐glycosylation inhibitor and ER stress inducer (Yoon et al. 2023). After 16 h of treatment, expression of all antigen variants was clearly reduced in transgenic cells; however, no significant change in molecular weight was observed (Figure 1c). This result strongly suggests that the presence of the CA1 signal peptide, rather than N‐glycosylation, is responsible for the observed size difference between the untreated HBV antigens. The decrease of antigen accumulation in TM‐treated cells can be accounted for by the unfolded protein response (UPR) and the ER‐associated protein degradation (ERAD) triggered by ER stress in the presence of the protein N‐glycosylation inhibitor (Wang et al. 2015). This result also suggests that HBVAg* is likely translocated into ER in the absence of the CA1 signal peptide, implying that the intrinsic TMD‐located ER translocation/anchor signals are functional in red alga cells.

The immunogenic properties of the HBV surface proteins strongly depend on their ability to form VLPs exposing repetitive protein structures. Since the presence of C‐terminal tags was previously shown to hinder the assembly of HBV antigens into VLPs (Pastor et al. 2019), which was confirmed in this study by sucrose gradient ultracentrifugation (Figure S1), we additionally constructed an untagged version of the HBVAg that also lacked the CA1 signal peptide (Figure 1a) and introduced it into *P. purpureum* by transformation. Several transgenic lines expressing the untagged HBVAg (1–8) were obtained (Figure 1d), and antigen accumulation was verified by immunoblotting using a commercial HBV‐S antibody (Figure 1e). All tested lines accumulated the HBVAg to much higher levels than either of the HBVAg* variants, suggesting a more efficient synthesis and/or stability of the untagged version.

To generate a sufficient amount of the antigen for characterisation and animal immunisation experiments, the strongest expressing line of the untagged HBVAg antigen (Figure 1d, lane 4) was grown in a custom‐made photobioreactor (Hammel, Cucos, et al. 2024). After inoculation with 1 × 10^5^ cells/mL, the cultures were grown for 8 to 13 days to the mid‐exponential phase. In total, 8 L of culture were grown to an average cell density of 4.4 ± 2.2 × 10^6^ cells/mL and an average dry weight of 0.4 ± 0.2 mg/mL, amounting to 3 g dry transgenic *Porphyridium* biomass which was used for HBVAg VLP purification. The total protein content of the transgenic strain as a percentage of the dry weight was determined by total protein extraction with phenol and BCA measurement. This analysis revealed that approximately 22% of the dry algal biomass is protein.

Quantification of the PBR culture‐derived antigen was performed by western blot analysis using HBV‐S antibodies and employing a recombinant HBV‐S protein as a standard. These assays revealed an antigen accumulation level of ~3.6% of the total cellular protein (TP; Figure 1e). Taking the TP amount per dry weight into account, 1 g of dried biomass derived from the untagged HBVAg‐expressing cells contains approximately 8 mg chimeric HBV antigen. The calculated volumetric yield was 3.2 mg antigen per litre of PBR‐grown culture.

### 2.2 Molecular Characterisation of HBVAg Produced in *P. purpureum*

**FIGURE 2:**
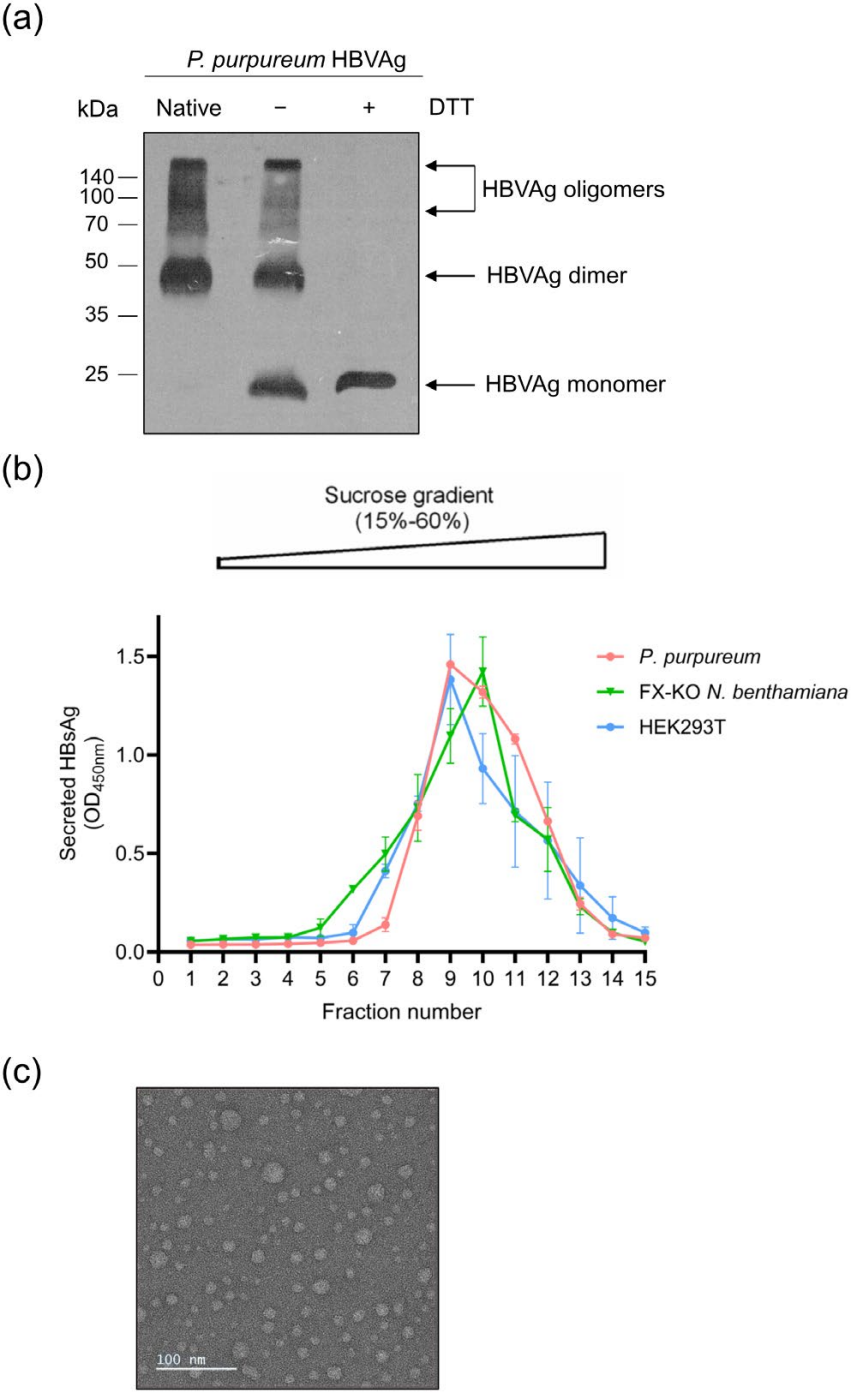
Molecular characterisation and assembly of the untagged HBVAg in *P. purpureum*. (a) Lysates of freeze‐dried algal biomass were subjected to SDS‐PAGE under native, non‐reducing (−DTT) and reducing (+DTT) conditions, and protein bands corresponding to HBVAg monomers, dimers and oligomers were detected by western blot analysis using anti‐preS1 antibodies. (b) HBVAg antigens produced in HEK293T cells, transiently transformed FX‐KO *N. benthamiana*, and *P. purpureum* were first concentrated via 20% sucrose ultracentrifugation and then separated by sucrose gradient (15%–60%) ultracentrifugation to determine the capacity of the antigens to assemble into high‐molecular weight VLPs (fractions 8–11). The presence of the HBVAg in the collected fractions was determined by ELISA. (c) Purified HBVAg was negatively stained with 2% uranyl acetate and imaged by TEM. Spherical particles with diameters of approximately 15 to 40 nm are seen, consistent with size and shape of previously observed chimeric HBVAg VLPs produced in mammalian cells. Scale bar: 100 nm.

For initial molecular characterisation, HBVAg extracted from freeze‐dried algal biomass was separated via SDS‐PAGE under native, non‐reducing (−DTT) and reducing (+DTT) conditions. We found that the *P. purpureum‐*produced antigen forms intermolecular disulfide bonds, as evidenced by the presence of protein bands at ~50 kDa under both native and non‐reducing conditions, corresponding to the expected size of protein dimers (Figure 2a). Moreover, protein bands of higher molecular weight were also detected, suggesting assembly into higher‐order oligomers. Our analysis also revealed the virtual absence of protein bands corresponding to the glycosylated form of the antigen (~27 kDa) that had previously been observed in mammalian cells (Pantazica et al. 2022). Currently, little information is available about protein N‐glycosylation in *P. purpureum* , although we have recently shown that the HCV‐E2 antigen produced in this organism contains short‐chain, high‐mannose glycans (Hammel, Cucos, et al. 2024). Given that N‐glycosylation is not required for HBsAg assembly in mammalian cells (Julithe et al. 2014), the potential lack of N‐glycans should not affect the assembly of the HBVAg into VLPs in *P. purpureum* . To verify this hypothesis, algal lysates were subjected to sucrose‐gradient ultracentrifugation, followed by detection of antigen‐containing fractions by ELISA. For comparison, antigen samples from mammalian cells and transiently transformed FX‐KO *N. benthamiana* plants were also tested. These analyses revealed that the HBVAg displayed similar sedimentation rates regardless of the production system, strongly suggesting that red algal cells support the faithful assembly of the chimeric antigen into high‐molecular weight VLPs (Figure 2b).

To further confirm the formation of virus‐like particles, we performed transmission electron microscopy (TEM) on purified HBVAg fractions. As shown in Figure 2c, the samples contained spherical particles that were slightly heterogeneous in size, ranging from 15 to ~40 nm in diameter. Particle size and morphology are consistent with those of previously observed chimeric HBVAg VLPs produced in mammalian cells (Dobrica et al. 2017). These results confirm that the algal‐expressed HBVAg assembles into VLPs.

### 2.3 Antigenic Properties of HBVAg VLPs Produced in *P. purpureum*

**FIGURE 3:**
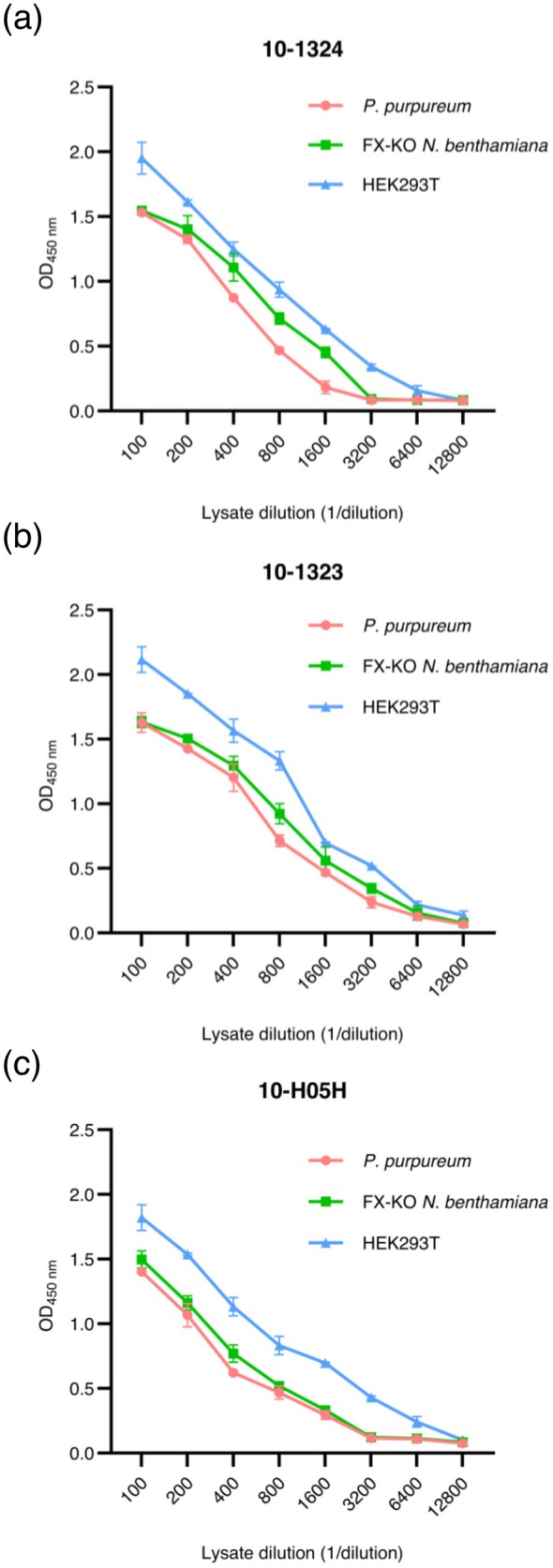
Antigenicity of HBVAg produced in *P. purpureum* . (a–c) A set of three conformation‐dependent, monoclonal anti‐S antibodies was used to coat 96‐well plates, followed by incubation with serial dilutions of equal quantities of *P. purpureum* , FX‐KO *N. benthamiana* or HEK293T‐produced HBVAg VLPs, as quantified via western blot analysis with anti‐preS1 antibodies. The plates were incubated with a secondary rabbit anti‐S antibody, followed by exposure to an HRP‐conjugated anti‐rabbit antibody and the corresponding substrate. Antigen binding is shown as optical density values (OD) measured at 450 nm. Data are represented as mean ± SD (*n* = 4).

To further characterise the antigenic features of the *P. purpureum* ‐derived HBVAg, we evaluated its binding to a panel of commercially available, conformation‐dependent anti‐S antibodies. Antigen samples from mammalian cells and transiently transformed FX‐KO *N. benthamiana* plants were also tested for comparison. These antibody‐binding experiments were performed using antigen fractions that contained only high‐molecular‐weight VLPs. The results indicated that the HBVAg produced in HEK293T cells was recognised by the anti‐S conformational antibodies slightly more efficiently than the antigen produced by plant and algal cells (Figure 3). This is unsurprising, given that the anti‐S antibodies were obtained by immunisation with viral particles purified from mammalian cells. Interestingly, the *P. purpureum‐*derived HBVAg showed similar binding properties as the protein transiently produced in FX‐KO *N. benthamiana* leaves, which had been shown previously to elicit a strong humoral immune response in mice and induce virus‐neutralising antibodies (Pantazica et al. 2023). Although subtle differences in antigen conformation depending on the production platform cannot be excluded, the efficient anti‐S antibody binding of the *P. purpureum‐*produced antigen, together with the ability of the antigen to form VLPs, warranted further testing of its immunogenic properties in vivo.

### 2.4 Purification of HBVAg From *P. purpureum*

**FIGURE 4:**
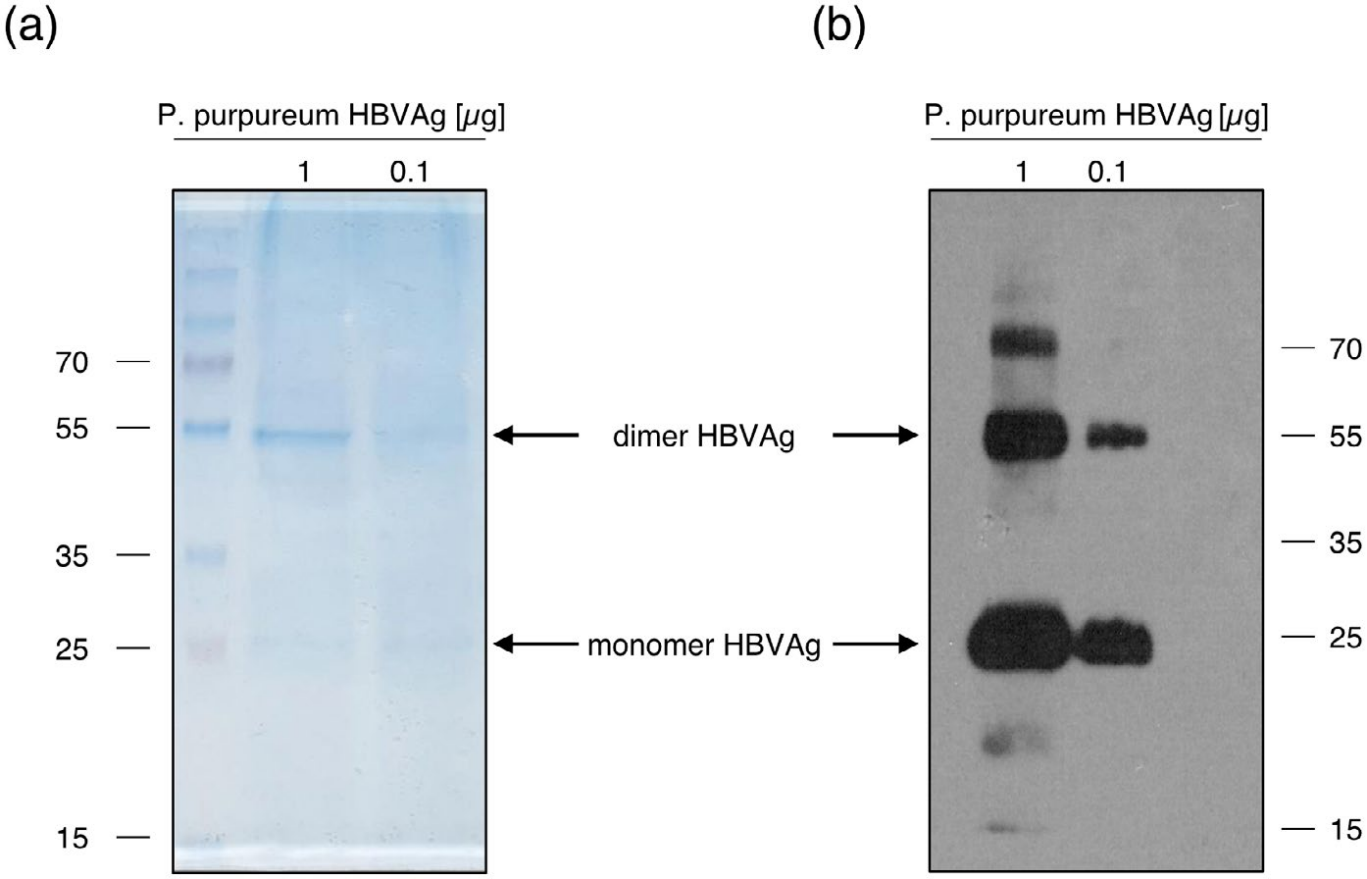
Purification of HBVAg expressed in *P. purpureum* . Freeze‐dried algae were first subjected to osmotic shock by incubation in ddH_2_O overnight, followed by centrifugation and collection of the supernatant which contained the antigen. The remaining algal pellet was further lysed using protein lysis buffer to extract all remaining antigen. Samples were then 20‐fold concentrated, followed by fractionation on a 15%–60% sucrose gradient. Antigen‐positive fractions were collected and subjected to anion exchange purification, followed by gel filtration. Samples were concentrated and analysed by Coomassie staining (a) and western blot analysis (b) using mouse anti‐preS1 monoclonal antibodies.

Several purification methods were evaluated for large‐scale purification of the HBVAg from *P. purpureum*. As addition of the 3xHA‐tag negatively affected both antigen expression levels (Figure 2b) and VLP assembly (Figure S1), purification using affinity tags could not be considered. Another major challenge was the presence of large amounts of phycoerythrin (PE) in microalgae cell extracts. PE is one of the most abundant light‐harvesting pigment‐protein complexes in red algae and cyanobacteria (Lee et al. 2021). PEs (and other phycobiliproteins) are water‐soluble protein complexes that accumulate to high levels in the chloroplasts of red algae (Li et al. 2019). To efficiently remove them from algal cells while preserving the structure and function of the VLP‐assembled HBVAg, we first used osmotic shock for cell disruption, by incubating freeze‐dried algal biomass with deionised water. Analysis of the soluble extracts by Coomassie staining and western blotting showed that a significant amount of antigen was released together with the PE by this procedure (Figure S2). To further purify the antigen, samples were first concentrated by ultracentrifugation through a 20% sucrose bed and then separated by ultracentrifugation in a 15%–60% sucrose gradient to enrich VLPs. However, fractions positive for the HBVAg also contained high amounts of PE, likely due to PE forming large oligomeric protein complexes (trimers and hexamers) that assemble the phycobilisome (Li et al. 2023). Therefore, an additional ion‐exchange purification step was performed to remove the pigment‐protein complexes, based on the predicted differences in charge between PE and the HBVAg (Figure S3). Finally, residual contaminating PEs were removed by gel‐filtration using a CaptoCore 400 resin with a cut‐off of ~300 kDa (Figure S4). In addition, to recover any remaining antigen from the algal pellet that had been obtained by centrifugation after the initial osmotic shock extraction, the pellet was treated with lysis buffer containing 0.25% Triton‐X and a protease inhibitor cocktail, and the resulting protein extract was subjected to gel filtration on the CaptoCore400 resin. Antigen‐containing fractions from both the soluble extract and the pellet were pooled, concentrated and separated by SDS‐PAGE, followed by Coomassie Blue staining (Figure 4a) and western blotting (Figure 4b). On the basis of these analyses, the recovered antigen yield was estimated to be approximately 0.75 μg/mg dry weight of algal biomass.

### 2.5 HBVAg Produced in *P. purpureum* Elicits Strong Humoral and Cellular Immune Responses in Vaccinated Mice

**FIGURE 5:**
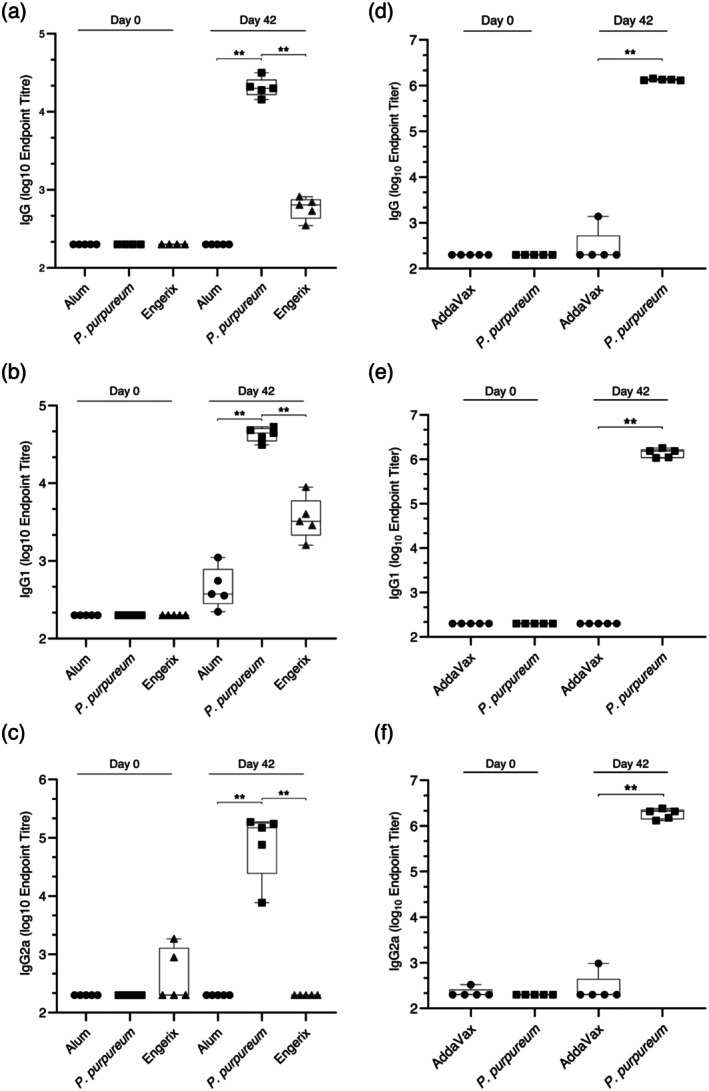
Humoral immune response triggered by the *P. purpureum* ‐produced HBVAg adjuvanted with Alum (a–c) or AddaVax (d–f). In the Alum‐adjuvanted vaccine trial (a–c), groups of five mice were immunised with adjuvant only (Alum, control), Engerix or Alum‐adjuvanted HBVAg. In the AddaVax‐adjuvanted trial (d–f), groups of five mice were immunised with AddaVax only (control), or AddaVax‐adjuvanted HBVAg produced in *P. purpureum* . Endpoint titers of IgG (a, d), IgG1 (b, e) and IgG2a (c, f) at 0 and 42 days post‐immunisation were calculated based on a 4‐parameter logistic regression curve fitted to a pool of immune sera. The values were calculated as the reciprocal of the sample dilution that resulted in a signal three times the baseline value ± standard error. The baseline value was derived from the internal standard curve through multiplication (*n* = 5). Statistical analysis was performed by using the Wilcoxon rank‐sum test (**p < 0.01).

To characterise the immunogenic properties of the alga‐produced antigen and its potential suitability as an HBV vaccine, BALB/c mice were immunised with either *P. purpureum* ‐produced HBVAg or, as a positive control, with Engerix B, the commercially available HBV vaccine containing the recombinant S protein produced in yeast. Since the HBV antigen is adjuvanted with aluminium hydroxide (Alum) in the commercial vaccine, the same adjuvant was used for mice immunisation with the alga‐produced antigen. A control group immunised with Alum only was also included (Figure 5a–c).

Our previous work had indicated that oil‐in‐water emulsion adjuvants such as AddaVax may enhance the efficiency of HBV vaccine candidates by inducing a faster and stronger immune response when compared to Alum (Pantazica et al. 2023; Pulendran et al. 2021). To substantiate this observation, a group of mice immunised with AddaVax‐adjuvanted *P. purpurem*‐produced HBVAg was also included in the experiment (Figure 5d–f). Investigation of the immune responses in the vaccinated animals indicated that the Alum‐adjuvanted HBVAg elicited significantly higher titers of total IgG than Engerix B (Figure 5a). Interestingly, this increase was based on a balanced secretion of both the IgG1 and IgG2a subclasses, indicating similar activation of Th2 (humoral) and Th1 (cellular) immune responses (Figure 5b,c). By contrast, Engerix B triggered only an IgG1‐based humoral immune response (Figure 5b,c), as expected for this type of Alum‐adjuvanted vaccines (Zhao et al. 2023). The use of Alum in both vaccines suggests that the antigen structure rather than the adjuvant is responsible for the difference in immune response activation. Notably, the same IgG1/IgG2a response, although of higher magnitude, was observed with the AddaVax‐adjuvanted HBVAg (Figure 5d–f). This result confirms that the Th1/Th2 cell activation pattern is a property of the chimeric HBV antigen, and moreover, supports the notion that oil‐in‐water adjuvants effectively potentiate the immune response to HBV vaccines.

**FIGURE 6:**
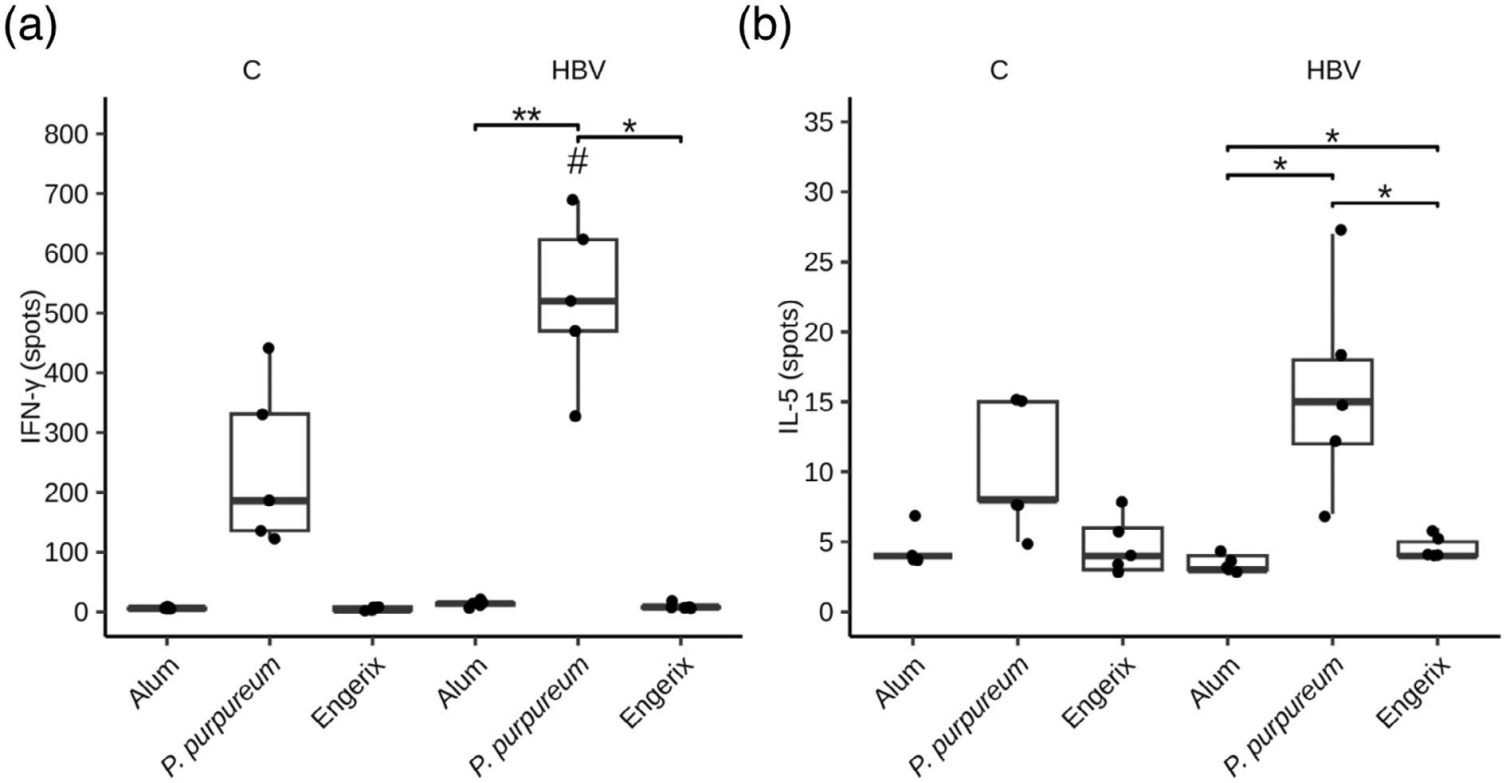
Cellular immune responses induced by the *P. purpureum* ‐produced HBVAg adjuvanted with Alum. Groups of five mice were immunised with adjuvant alone (Alum), *P. purpureum* ‐produced HBVAg adjuvanted with Alum ( *P. purpureum* ), or a commercially available HBV vaccine (Engerix). Cytokine levels of IFN‐γ (a) and IL‐5 (b) were assessed in spleen cells harvested on day 42 by ELISpot assays. Spleen cells were stimulated with UV‐inactivated HBV (HBV) and compared to non‐stimulated control cells (C). Statistical analysis was performed by using the Wilcoxon rank‐sum test. Comparisons between the HBV‐stimulated groups (*p < 0.05; **p < 0.01), and between the HBV‐stimulated and their non‐stimulated (C) controls (^#^ *p* < 0.05) are shown.

To gain more insights into the cellular immune response induced by the *P. purpureum‐*produced HBV antigen, spleen cells from mice immunised with either Alum‐adjuvanted HBVAg, Engerix B vaccine or Alum alone were stimulated with UV‐inactivated HBV. Unstimulated cells provided the baseline level of cell activation. Secretion of IFN‐γ and IL‐5, signature cytokines of Th1 and Th2 cell‐type activation, respectively, was determined by ELISpot (see Experimental Procedures). HBV stimulation significantly increased the number of IFN‐γ and IL‐5‐secreting spleen cells from mice that had received the *P. purpureum* ‐produced HBVAg, as compared with the adjuvant‐only and the Engerix group (Figure 6a,b). The relatively low magnitude of the IL‐5 response suggests that immunisation with the red alga‐produced HBVAg may favour a Th1 cell‐type activation in mice. By contrast, no cellular immune response could be detected in our experimental setup following Engerix B immunisation (Figure 6), consistent with the absence of IgG2a secretion observed before (Figure 5c).

### 2.6 Immunisation With HBVAg Produced in *P. purpureum* Induces Antibodies With Potent Neutralisation Activity Against Both Wild‐Type and VEM HBV

**FIGURE 7:**
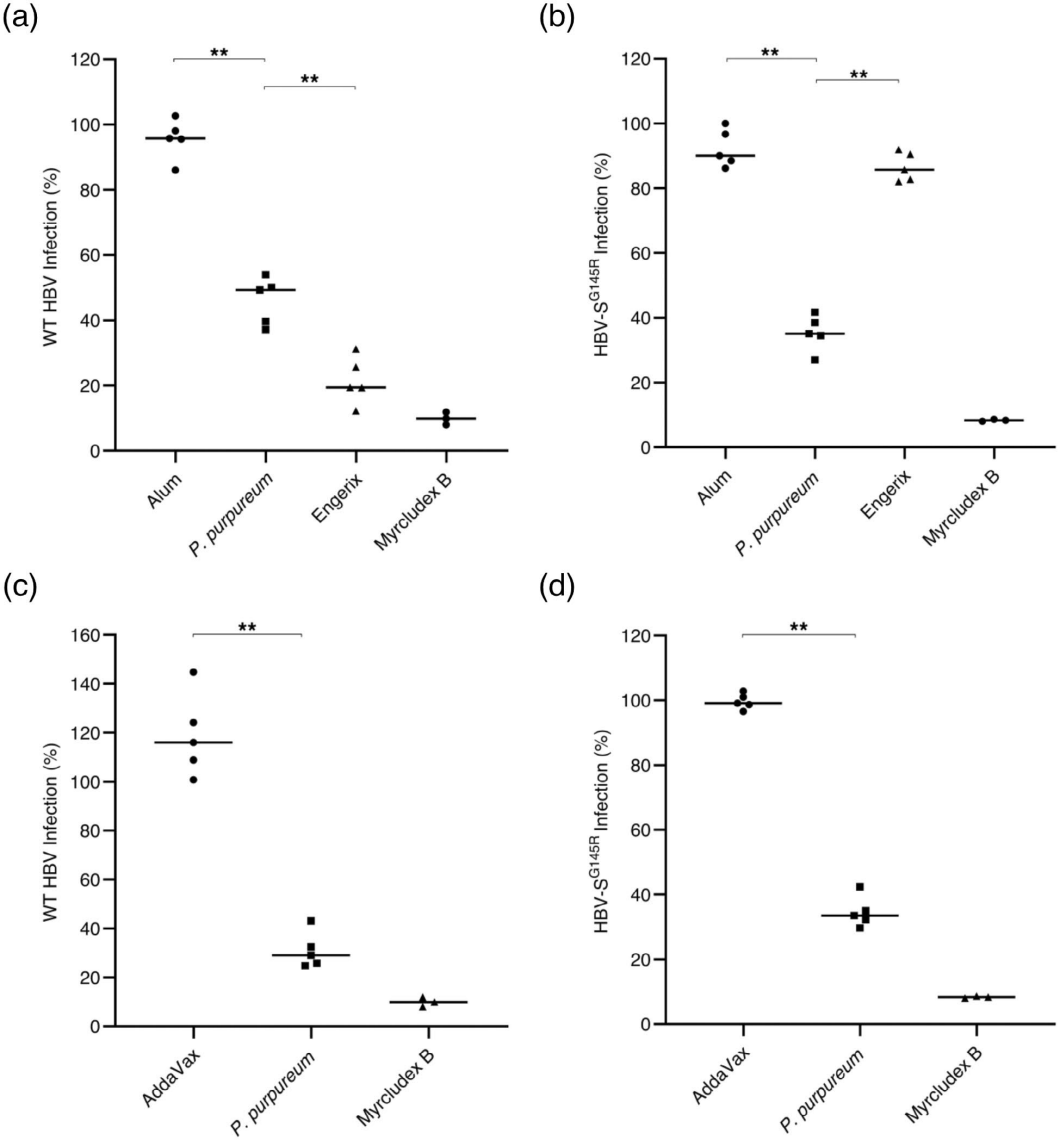
Neutralisation capacity of sera from mice immunised with HBVAg expressed in *P. purpureum* against wild‐type (WT) HBV and VEM HBV (HBV‐S^G145R^). Sera from mice immunised with HBVAg produced in *P. purpureum* adjuvanted with Alum (a, b) or AddaVax (c, d), the current commercial vaccine Engerix (a, b), and the adjuvant‐only control were diluted 1:50 and pre‐incubated with WT (a, c) or VEM (b, d) HBV inoculum (100 Geq/cell), in medium supplemented with 4% PEG, for 1 h. HepG2^hNTCP^ cells were incubated with the serum‐treated HBV inoculum for 16 h. Cells incubated with Myrcludex B for 3 h prior to infection were used as a control for the specificity of inhibition of HBV infection. At 16 h post‐infection, the medium was supplemented with 2.5% DMSO and then exchanged every 2 days. Cell media were collected at day 11 post‐infection, and used to quantify the HBeAg levels by ELISA. The data shown represent HBV infection rates in the presence of post‐immune serum relative to infection rates in the presence of the pre‐immune serum (at the same dilution), in percentages. Values in the presence of Myrcludex B represent infection rates relative to HBV‐only samples (as percentages). Statistical analysis was performed with the Mann–Whitney *U* test (**p < 0.01).

An increased antibody titre following immunisation of mice with HBVAg is not necessarily indicative of efficient inhibition of HBV infection. Therefore, to evaluate the quality of the raised immune response, we compared the infection‐neutralising capacity of antibodies induced by immunisation with Alum‐adjuvanted *P. purpureum* ‐derived HBVAg or Engerix B against both WT HBV and a clinically relevant VEM variant, HBV‐S^G145R^ (Pantazica et al. 2022; Zuckerman 2000). To this end, WT and VEM HBV inocula were treated with pre‐immune or post‐immune sera (1:50 dilution) collected from mice vaccinated with either the yeast‐derived or the alga‐derived antigen (or an adjuvant‐only control), and then used to infect HepG2^hNTCP^ cells at 100 GEq/cell. Cells pretreated with Myrcludex B, a highly selective HBV entry inhibitor, were used as an additional control. HBV infection was monitored by measuring secreted HBeAg using ELISA, and the results were normalised to the HBeAg levels observed in the presence of pre‐immune serum. Importantly, immune sera derived from mice receiving *P. purpureum‐*produced antigen showed strong virus neutralisation activity against both WT (Figure 7a) and HBV‐S^G145R^ (Figure 7b), consistent with what was previously observed for the chimeric antigen produced in mammalian cells (Pantazica et al. 2022). Engerix B was more potent than the *P. purpureum* ‐derived HBVAg in inducing a humoral immune response with neutralising activity against the WT HBV. This is not unexpected, considering the higher purity of the HBV antigen in Engerix B and the established clinical efficiency of this vaccine since it has been introduced into the market (Schillie et al. 2018). However, the chimeric antigen produced in *P. purpureum* was significantly more effective in neutralising the clinically relevant VEM HBV variant when compared to Engerix. This is consistent with previous data showing the absence of neutralising antibodies against VEM HBV following immunisation with the S protein (Pantazica et al. 2022).

The immune sera from mice immunised with AddaVax‐adjuvanted HBVAg also displayed significant neutralisation capacity against both WT and VEM HBV (Figure 7c,d), validating the robust immunogenicity of the alga‐derived antigen. Direct comparison of HBV neutralisation capacity of sera from mice immunised with the HBVAg in the presence of either Alum or AddaVax confirmed the superior capacity of the latter adjuvant to potentiate the immunogenic properties of the antigen (Figure S5).

## 3 Discussion

Poor response to available HBV vaccines remains a major public health concern, especially in highly endemic areas. Multiple approaches, including novel antigen designs and production platforms, improved vaccine adjuvants and enhancement of the innate immune response, have been employed in the last decades to address the urgent need for better vaccines (Gerlich 2017). The first approved 3‐antigen HBV vaccine that contained all three viral envelope proteins (S, M and L) has been shown to induce protective antibody levels in non‐responders to the S‐only vaccines (Vesikari, Finn, et al. 2021; Vesikari, Langley, et al. 2021). However, due to the complex folding requirements of the HBV‐M and L proteins and their strong interference with ER homeostasis when they are overexpressed, these protein cocktail‐based vaccines can only be produced in highly cost‐intensive mammalian cell cultures.

To tackle the limitations of current vaccines, we have proposed an alternative HBV vaccine design that uses the S protein as both a structural scaffold for VLP assembly and a carrier for L‐derived immunogenic peptides (Dobrica et al. 2017). The chimeric S/L antigens assemble into highly immunogenic VLPs in mammalian cells and plants, and trigger both anti‐S and anti‐L antibodies with potent HBV‐neutralising activity (Dobrica et al. 2017; Pantazica et al. 2022). Thus, combining appropriate immunogenic epitopes in a single molecule, rather than using entire proteins, may constitute a valid strategy for the development of improved HBV vaccine candidates that are amenable to production in cost‐efficient biotechnological systems. Moreover, the ability of the HBV‐S protein to accept foreign peptides derived from other important human pathogens such as poliomyelitis virus, malaria‐causing protozoa or HCV may enable the development of advanced biotechnological platforms for the production and delivery of new, medically relevant antigens (Ho et al. 2020).

Owing to their ability to grow at rates that are similar to bacterial cells, while at the same time providing all the advantages of eukaryotic expression systems (e.g., post‐translational modifications, assembly of multimeric protein complexes), microalgae have been considered as potential production hosts for the synthesis of the HBV‐S protein, the major constituent of commercial HBV vaccines (Geng et al. 2003; Hempel et al. 2011). However, the reported levels of HBV‐S protein expression achieved in the microalgal species explored so far, the green alga *Dunaliella salina* and the diatom *Phaeodactylum tricornutum* , are very low: ~3 ng/mg of TSP and ~0.7% of TSP, respectively (Geng et al. 2003; Hempel et al. 2011). Moreover, the poor yields have precluded a thorough investigation of the immunogenic properties and the ability of the antigen to assemble into VLPs.

In this work, we have demonstrated the potential of the red alga *P. purpureum* to support the synthesis of a chimeric HBV S/L antigen, previously developed in mammalian cells and transiently expressed in *N. benthamiana* leaves (Pantazica et al. 2022, 2023). Our data revealed unprecedented efficiency of antigen production in the red alga, reaching ~8 mg/g of dried biomass, and accounting for ~3.6% of the total protein content upon algal cultivation in a PBR. Notably, this amount is approximately 10 times higher than that of the HCV‐E2 antigen produced in *P. purpureum* (0.5–0.89 mg/g dry weight; Hammel, Cucos, et al. 2024). This remarkable difference is likely due to the property of the HBV antigen to self‐assemble into stable VLPs, preventing viral protein aggregation and ER stress, and potentially reducing the susceptibility to proteolytic degradation. Indeed, the *P. purpureum* antigen was efficiently translocated into the ER in the absence of alga‐specific ER‐targeting signal sequences. Moreover, it oligomerised by disulfide bond formation and assembled into VLPs, indicating that the factors controlling these processes are functional in the red alga.

Despite the high accumulation levels of the HBVAg in *P. purpureum* , we could not detect antigen secretion into the culture medium in the transgenic algal strains that targeted the antigen to the secretory pathway (Figure 1). This is likely due to the properties of the HBVAg, given that the soluble HCV‐E2 antigen is secreted from *P. purpureum* cells, albeit at low levels (Hammel, Cucos, et al. 2024). Interestingly, the intracellular retention of the HBV antigen and the apparent lack of glycosylation of the conserved N‐glycan site in the red alga are reminiscent of the expression of HBV surface proteins in yeast, and had no obvious negative consequences on the production of highly immunogenic HBVAg VLPs. More work will be needed to better understand the molecular details of HBV VLPs trafficking along the secretory pathway in algal cells, and to establish whether efficient extracellular antigen secretion can be achieved in microalgae.

Our present study also provided the first evidence of the in vivo immunogenicity of an HBV vaccine candidate expressed in red algae. Immunisation of mice with the *P. purpureum* ‐derived HBVAg triggered strong humoral and cellular immune responses superior to those induced by a commercial vaccine. This was evidenced by the pattern of IgG1/IgG2a secretion and the activation of both IFN‐γ and IL‐5‐secreting spleen cells. Moreover, strong inhibition of cell infection with either WT or a clinically relevant VEM HBV was observed in the presence of immune sera from HBVAg‐vaccinated mice. These findings suggest that, similar to the antigen produced in mammalian cells, the *P. purpureum‐*derived antigen presents key conformational epitopes to the immune system that elicit a strong, virus‐neutralising antibody response. As observed in previous studies (Pantazica et al. 2023), this response was more efficiently potentiated by AddaVax, indicating that oil‐in‐water adjuvants are more appropriate than Alum for use in vaccines against HBV.

To preliminarily assess the practical utility of our production system, we estimated the volume of *P. purpureum* culture required to generate vaccine‐relevant quantities of antigen. On the basis of our current yield and a standard human vaccine dose of 20 μg/dose (as used in licensed HBV vaccines), we estimate that 1 L of algal culture would yield sufficient antigen for approximately 15 doses, enough to fully immunise 3 individuals using the standard three‐dose schedule. However, further optimisation of purification protocols and process scale‐up may substantially reduce this volume requirement and enhance the economic viability of the system.

In summary, by demonstrating successful production of a complex viral antigen and assembly of highly immunogenic VLPs, our study illustrated the potential of microalgae as versatile protein production factories and suggests *P. purpureum* as a promising new platform for the large‐scale synthesis of recombinant proteins of biotechnological interest for human and animal health.

## 4 Experimental Procedures

### 4.1 Cell Lines, Plasmids and Virus Production

HEK293T and Huh7 cells were maintained in Dulbecco's Modified Medium (DMEM) supplemented with 10% fetal bovine serum (FBS) and 1% non‐essential amino acids (NEAA). HepG2^hNTCP^ cells (gift from Professor Stephan Urban, German Center for Infection Research, University of Heidelberg) were cultured and used for HBV neutralisation assays as previously described (Pantazica et al. 2022). HepG2.2.2.15 cells stably transfected with two copies of the HBV genome (gift from Dr. David Durantel, INSERM U871, Lyon, France) were used for the production of WT‐HBV stocks, as previously reported (Dorobantu et al. 2011). All cell lines were maintained at 37°C in an incubator supplied with 5% CO_2_. Stocks of clinically relevant VEM, containing the G145R mutation within the antigenic domain of S, were obtained by transfection of Huh7 cells with pGEM‐4Z‐HBV 1.3 G145R (Pantazica et al. 2022) by using Lipofectamine 3000 (Invitrogen) according to the manufacturer's protocol. Cell media were collected between days 7 and 12 post‐transfection, concentrated by ultracentrifugation on a 20% sucrose layer, and quantified by real‐time qPCR (Lazar et al. 2007). For antigenicity tests, HEK 293T cells were transfected with a pCi plasmid encoding the S/preS1^16‐42^ antigen, as previously described (Pantazica et al. 2022).

### 4.2 Cultivation and Transformation of *P. purpureum*

Cultivation of *P. purpureum* strain SAG 1380‐1d (obtained from the Culture Collection of Algae at the University of Göttingen, Germany) was performed under photoautotrophic growth conditions in low‐salt artificial seawater (lsASW) medium (Hammel, Neupert, and Bock 2024). Cultures were maintained under continuous light (100 μmol photons m^−2^ s^−1^) with agitation at 120 rpm. For experiments with the N‐glycosylation inhibitor tunicamycin, 1 μg/mL of the compound was added to a culture with a cell density of 4.5 × 10^6^ cells/mL, and the culture was grown as described above. Cell numbers were determined using the Z2 Coulter Particle Count and Size Analyser (Beckman Coulter) by mixing 100 μL cell culture with 9.9 mL counting buffer (Isoton II Dilluent, Beckman Coulter). For cell number determination, cell diameters from 3.5 to 12 μm were considered, and all samples were counted twice (and the average value was used). Nuclear transformation was performed using the biolistic method as previously described (Hammel, Neupert, and Bock 2024).

### 4.3 Construction of Transformation Vectors

Plasmid pZL22 (Li and Bock 2018) harbouring the zeocin resistance gene *ble* under the control of the *P. purpureum* tubulin promoter and terminator was used to construct *P. purpureum* transformation vectors, as previously described (Hammel, Neupert, and Bock 2024). The coding sequence for the HBV‐S/preS1^16‐42^ antigen was codon optimised for nuclear expression in *P. purpureum* , sequences for the signal peptide of the endogenous carbonic anhydrase (at the N‐terminus) and the 3xHA‐tag, followed by a HDEL ER retention signal (at the C‐terminus) were added, and the gene was chemically synthetised (GeneCust). For cloning, the coding region of the chloroplast‐targeted YFP from pZL22 was excised using the restriction endonucleases XhoI and NheI, and replaced with the respective HBV antigen gene variant (amplified by PCR from the synthetised gene) via Gibson assembly. All primers used in this study are listed in Table S1.

### 4.4 Total Protein Extraction From Algal Cells

Total cellular proteins were extracted from cultured algal cells using a modified phenol/methanol method (Chatterjee et al. 2012). Briefly, algal cells were resuspended in protein extraction buffer containing 30% sucrose, 2% SDS, 10 mM Tris pH 8.0, 2% β‐mercaptoethanol and 1× cOmplete protease inhibitor (EDTA‐free; Roche), and then sonicated for 3 × 10 s at 20% amplitude on ice using a W‐250 D Sonifier (G. Heinemann, Schwäbisch Gmünd, Germany), and mixed with an equal volume of phenol (pH 7.6). After vigorous vortexing, the samples were centrifuged at 12 000 *g* at 4°C for 10 min. The upper phenolic phase was then collected, and proteins were precipitated by overnight incubation at −20°C with 4 volumes of ice‐cold 0.1 M ammonium acetate in 100% methanol. Following centrifugation at 12 000 *g* at 4°C for 5 min, the protein pellet was washed twice with ice‐cold methanol and resuspended in protein buffer containing 50 mM Tris HCl pH 7.6, 100 mM NaCl, 10 mM KAc, 5 mM MgAc, 0.5% SDS and 1× cOmplete protease inhibitor. Protein concentrations were determined via the BCA assay (Thermo Fisher Scientific).

### 4.5 Generation of *P. purpureum* Biomass by Photobioreactor (PBR) Culture

The PBRs used in this study (LWS‐05) were constructed by the Institute für Getreideverarbeitung (IGV GmbH, Nuthetal, Germany). Cultivation of *P. purpureum* was performed as described by Hammel, Cucos, et al. (2024) with the following changes: The PBR tube was aerated with pressured air, without the addition of CO_2_. The cultivation period was 9 to 12 days, depending on the run and the cell density, and the culture was harvested at cell densities of 4 to 8 million cells/mL.

### 4.6 SDS‐PAGE and Western Blot Analyses

Protein samples were heat‐denatured in Laemmli buffer under reducing (+25 mM DTT) or non‐reducing (without DTT) conditions, and then separated by SDS‐PAGE using either 10% or 12% polyacrylamide (PAA) gels. For analysis of antigen expression, the electrophoretically separated proteins were blotted onto nitrocellulose membranes, followed by incubation with either mouse anti‐HA (GeneScript; A01244) or rabbit anti‐HBV‐S antibodies (Novus Biologicals; NB100‐62652, diluted 1:1000 in PBS containing 5% non‐fat milk and 0.1% Tween), for 1 h at room temperature (RT). Detection of purified HBV‐S/preS1 antigens was performed by incubation with mouse monoclonal anti‐preS1 antibodies (Santa Cruz Biotechnology, sc‐57762, diluted 1:1000 in PBS containing 1% non‐fat milk and 0.1% Tween), overnight at 4°C. Following three washes with 0.1% Tween in PBS, membranes were incubated with HRP‐labelled anti‐mouse antibodies (Agrisera; AS111772, diluted 1:25 000 in PBS containing 5% non‐fat milk and 0.1% Tween) or HRP‐labelled anti‐rabbit antibodies (Bio‐Rad; 170‐6515, diluted 1:10 000 in PBS containing 5% non‐fat milk and 0.1% Tween). For quantification of the purified HBV‐S/preS1 antigen, HRP‐conjugated mouse IgGκ‐binding protein (Santa Cruz Biotechnology, sc‐516102, diluted 1:10 000 in PBS containing 5% non‐fat milk and 0.1% Tween) was used as secondary antibody. In‐house expressed 3xHA‐tagged YFP and a recombinant HBV S protein (Jena Bioscience, PR‐1197) were used as standards for semiquantitative assessment of the 3xHA‐tagged and the untagged antigen, respectively. The concentrations of the purified antigen were estimated using a standard curve of commercial L protein (preS1 antigen, Beacle) processed under the same experimental conditions (Dobrica et al. 2017).

### 4.7 Antigen Extraction From *P. purpureum*

Freeze‐dried algal biomass was first incubated in double‐distilled water (ddH_2_O) (1 mL ddH_2_O per 10 mg algae) at 4°C overnight with head‐over‐tail agitation to induce osmotic shock. The mixture was then centrifuged at 10 000 *g* for 15 min, and the supernatant was retained as the osmotic shock fraction. The remaining pellet was lysed in lysis buffer containing 150 mM NaCl, 20 mM Na_2_HPO_4_, 20 mM sodium ascorbate, 0.25% Triton‐X (pH 7.2) and a protease inhibitor cocktail (1×) for 30 min on ice, followed by centrifugation at 10 000 *g* for 15 min. The supernatant was retained as the lysate fraction. Samples resulting from both osmotic shock and pellet lysis were then 20‐fold concentrated via ultracentrifugation on a 20% sucrose bed at 32 000 rpm (SW 32 Ti, Beckman Coulter) for 5 h at 4°C. HBV‐S/preS1^16‐42^ VLPs were then isolated via sucrose gradient ultracentrifugation (15%–60%) at 30 000 rpm (SW 42Ti, Beckman Coulter) for 16 h. Fractions were collected and tested for the presence of HBV antigen via ELISA using the Monolisa HBsAg Ultra kit (BioRad) according to the manufacturer's instructions. Positive fractions were pooled and dialysed against 20 mM Na_2_HPO_4_ (pH 7.2) for further purification.

### 4.8 Purification of HBV‐S/preS1^16^ ^‐42^ Antigen Produced in *P. purpureum*

The HBV‐S/preS1^16‐42^ antigen produced in *P. purpureum* was purified via ion‐exchange chromatography, followed by gel filtration using the CaptoCore 400 resin (Pantazica et al. 2022). Briefly, the column (HiTrap Q HP column, Cytiva) was washed with 5 column volumes (CV) of start buffer (20 mM sodium phosphate, pH 7.2) before sample application. The HBV‐S/preS1 antigen was dialysed against the start buffer, and then loaded onto the column at a flow rate of 1 mL/min. The column was washed with 15 CV of start buffer, followed by elution using a linear NaCl gradient (0–500 mM). Flow‐through, wash, and elution fractions were collected and tested for the presence of the HBV‐S/preS1 antigen by western blotting using anti‐preS1 antibodies (Santa Cruz Biotechnology, sc‐57762) and ELISA using the Monolisa HBsAg Ultra kit (BioRad). The fractions containing the antigen were dialysed against PBS and further purified by gel‐filtration using the CaptoCore 400 resin (Cytiva) as previously described (Pantazica et al. 2022). Positive fractions were concentrated using 100 kDa cut‐off Amicon columns (Millipore, Sigma‐Aldrich) and analysed by SDS‐PAGE, followed by Coomassie Blue staining or western blotting using anti‐preS1 antibodies.

### 4.9 Transmission Electron Microscopy (TEM)

For electron microscopic analysis, a droplet of a suspension of purified HBVAg was deposited on a glow‐discharged carbon‐coated copper grid. The grid was negatively stained with a 2% uranyl acetate (aqueous solution). TEM observation was carried out using a JEM F200 transmission electron microscope (JEOL Ltd. Japan), operated at 200 kV. The images were recorded on a Gatan Metro detector (Ametek Inc., Berwyn, PA).

### 4.10 Antigenicity of HBV‐S/preS1^16^ ^‐42^ Antigen Produced in *P. purpureum* by Sandwich ELISA

Production of the HBV‐S/preS1^16‐42^ antigen in HEK293T cells and *N. benthamiana* lines lacking β‐1,2 xylosyltransferase and α‐1,3 fucosyltransferase activity (FX‐KO) has been previously described (Pantazica et al. 2023). These proteins were also included in our study for comparison, to reveal potential host‐dependent antigenic properties. The supernatant of HEK293T cells and the plant and *P. purpureum* lysates expressing the HBV‐S/preS1 antigen were concentrated on a 20% sucrose bed via ultracentrifugation and further separated by size via sucrose gradient (15%–60%) ultracentrifugation. Antigen‐positive fractions were determined via ELISA and those corresponding to high‐molecular‐weight VLPs were pooled and dialysed against PBS. Plant samples were concentrated via SpeedVac (Thermo Scientific‐Pierce) centrifugation, and 100 kDa cut‐off Amicon columns (Millipore, Sigma‐Aldrich) were used to concentrate the mammalian cell and alga‐produced antigens.

To determine the reactivity of HBV antigens against known conformation‐dependent HBV antibodies, 96‐well MaxiSorp plates (Nunc Thermo Scientifc) were first incubated with 50 ng/well commercial anti‐S antibodies (#10‐H05H, #10‐1323, #10‐1324, Fitzgerald Industries International), overnight at 4°C. After extensive washing in PBS supplemented with 0.1% Tween‐20 (Sigma Aldrich), non‐specific binding sites were blocked by the addition of 10% dried skim milk for 1 h at RT. Equal amounts of HBV‐S/preS1 VLPs obtained from the three production systems, as quantified by immunoblotting with anti‐preS1 antibodies, were added to the plates and incubated for 3 h at 37°C, followed by extensive washing and incubation with a conformation‐independent rabbit‐anti‐S antibody (Novus Biologicals, NB100‐62652, 1:1000 in PBS containing 5% non‐fat milk and 0.1% Tween) for 1 h at 37°C. The plates were washed extensively again and then incubated with HRP‐conjugated anti‐rabbit antibodies (Santa Cruz Biotechnology, sc‐2357, 1:10 000 in PBS containing 5% non‐fat milk and 0.1% Tween) for 1 h at RT, followed by extensive washing and detection via incubation with the 3,3′,5,5′‐tetramethylbenzidine (TMB) substrate (BioRad) for 30 min. The enzymatic reaction was stopped by the addition of 2 N H_2_SO_4_, and the optical density (OD) at 450 nm was determined by using a Mithras Microplate Reader.

### 4.11 Animals and Immunisation

Animal experiments were conducted in accordance with standards set forth in the Council Directive 86/609/EEC and national legislation. The study was approved by the Ethical Committee of the ‘Cantacuzino’ Medico‐Military National Research Institute, number 374/10.12.2019, and the national designated authority, ANSVSA, number 488/22.01.2020. Five groups of female Balb/c mice (6–8 weeks old, *n* = 5/group) were immunised by intramuscular injection three times at a 14‐day interval with antigen‐adjuvant formulations at a final volume of 50 μL/dose. The immunisation protocol was divided into two experiments based on the adjuvant used: Alum, an aluminium hydroxide‐based adjuvant (Alhydrogel, InvivoGen, San Diego, CA), and AddaVax, an oil‐in‐water type of adjuvant (InvivoGen, San Diego, CA). In the first experiment, the groups of mice were immunised with either 20 μg/dose of *P. purpureum‐*produced HBV‐S/preS1^16‐42^ antigen adjuvanted with Alum, 20 μg/dose of a commercially available HBV aluminium‐adsorbed vaccine (Engerix B, GlaxoSmithKline Biologicals), or Alum adjuvant only as control. In the second experiment, the animal groups received either 20 μg/dose of *P. purpureum‐*produced HBV‐S/preS1^16‐42^ antigen adjuvanted with AddaVax or the adjuvant alone. Blood samples were collected from individual mice by orbital puncture prior to administration of the first dose and after the first and second boost at days 14 and 28, respectively. Blood sera were obtained from clotted blood following centrifugation at 14 000 *g* for 10 min at RT, then stored at −80°C until further use for antibody titration and analysis of virus‐neutralising capacity. The animals were sacrificed on day 42 under anaesthesia after the final bleed, and spleens were removed aseptically for analysis of the cellular immune response.

### 4.12 Analysis of Humoral Immune Response in Vaccinated Mice

Antigen‐specific IgG, IgG1 and IgG2a were detected via ELISA as previously described (Pantazica et al. 2022) using plates coated with a suspension of UV‐inactivated HBV (containing 0.6 μg/mL of HBsAg) in PBS. Comparison between groups and statistical analysis were performed by using the Wilcoxon rank‐sum test.

### 4.13 Analysis of the Cellular Immune Response in Vaccinated Mice

Antigen‐specific IFN‐γ and IL‐5 secreting cells were quantified by ELISpot, using the Mouse IFN‐γ/IL‐5 FluoroSpot kit (Mabtech, Sweden), as previously described (Pantazica et al. 2023). Fluorescence photographs were acquired on an AID iSpot FluoroSpot Reader (using the appropriate filters), and spot detection and counting were performed using the AID EliSpot software Version 7.0. Comparison between groups and statistical analysis were performed by using the Wilcoxon rank‐sum test.

### 4.14 Neutralisation of HBV Infection by the Immune Sera

Animal sera were collected at 42 days post‐immunisation. Samples were diluted 1:50 in complete DMEM medium supplemented with 4% polyethylene glycol (PEG, Sigma Aldrich) and incubated for 1 h at 37°C with the wild type or a clinically relevant HBV VEM variant, HBV‐S^G145R^ (100 genome equivalents/cell). Pre‐immune sera from individual mice in each group were pooled and diluted 1:50 prior to incubation with the virus. The viral inoculum was then used to infect HepG2^hNTCP^ cells seeded in 48‐well plates for 16 h at 37°C. Cells incubated with 1 μM Myrcludex‐B (Pepscan), an HBV entry inhibitor (Urban et al. 2014), for 3 h prior to infection were used as a control for specific inhibition of HBV infection. After infection, the cells were washed extensively with PBS to remove any remaining virus, and incubated in complete DMEM medium supplemented with 2.5% DMSO (AppliChem). Accumulation of the HBeAg was quantified in pooled cell media at days 7–11 post‐infection, using the Monolisa HBeAg‐Ab PLUS kit (BioRad). Inhibition of HBV infection by the immune sera is represented as a percentage from infection values in the presence of the pre‐immune sera, at the same dilution. Data were statistically analysed by using the Mann–Whitney *U* test in GraphPad Prism version 8.

## Author Contributions

R.B., N.B.N. and C.S. conceived the study, with J.L.C. involved in the discussions. R.B., N.B.‐N. and C.S. analysed data. A.‐M.P. produced the HBV antigen in mammalian cells and plants, carried out molecular analyses, and functional characterisation and purification of the *P. purpureum* ‐expressed HBV antigen, performed the HBV neutralisation studies and analysed data. A.H. constructed the vectors for antigen expression in algal cells, generated and analysed transgenic algal strains and performed the bioreactor experiments. M.M. performed the transmission electron microscopy. C.S. and A.O. designed the animal experiments and immunisation schedules. I.C., C.T., I.I. and C.S. performed the animal experiments. C.S., C.T., I.I. and A.O. obtained and analysed the immunological data. J.L.C. produced and provided the plant material expressing the HBV antigen. N.‐B.N., A.‐M.P., A.H., C.S. and R.B. wrote the manuscript. All other authors reviewed the manuscript.

## Conflicts of Interest

The authors declare no conflicts of interest.

## Supporting information


**Figure S1.** VLP assembly of HA‐tagged HBVAg produced in *P. purpureum* . Assembly into VLPs of HA‐tagged HBVAg produced in *P. purpureum* and untagged HBVAg produced in HEK293T and WT or FX‐KO *N. benthamiana* was verified by sucrose gradient ultracentrifugation.

**Figure S2.** Isolation of HBVAg produced in *P. purpureum*. Isolation of HBVAg VLPs produced in *P. purpureum* via osmotic shock followed by cell lysis and analysis of resulting extracts via Coomassie staining and western‐blot.

**Figure S3.** Purification of *P. purpureum‐*produced HBVAg by ion‐exchange chromatography. Anion exchange chromatography purification of HBVAg produced in *P. purpureum* and analysis of resulting fractions via Coomassie staining and western‐blot.

**Figure S4.** Purification of *P. purpureum‐*produced HBVAg by gel‐filtration on CaptoCore 400 resin. Size‐exclusion chromatography purification of HBVAg produced in *P. purpureum* and purified via anion exchange chromatography and analysis of resulting fractions via Coomassie staining and western blot.

**Figure S5.** Comparison between adjuvants (Alum vs. AddaVax) and their effects on the neutralisation capacity of sera from mice immunised with *P. purpureum* ‐expressed HBVAg. WT and VEM HBV neutralisation assays using sera from mice immunised with the *P. purpureum* ‐produced HBVAg in the presence of either Alum or AddaVax. Infection levels were quantified by ELISA.

**Table S1.** Primer sequences used for vector construction

## Data Availability

The data that support the findings of this study are available from the corresponding author upon reasonable request.

## References

[pbi70270-bib-0001] Banerjee, A. , and V. Ward . 2022. “Production of Recombinant and Therapeutic Proteins in Microalgae.” Current Opinion in Biotechnology 78: 102784. 36095993 10.1016/j.copbio.2022.102784

[pbi70270-bib-0002] Barbosa, M. J. , M. Janssen , C. Südfeld , S. D'Adamo , and R. H. Wijffels . 2023. “Hypes, Hopes, and the Way Forward for Microalgal Biotechnology.” Trends in Biotechnology 41: 452–471. 36707271 10.1016/j.tibtech.2022.12.017

[pbi70270-bib-0003] Bian, T. , H. Yan , L. Shen , et al. 2013. “Change in Hepatitis B Virus Large Surface Antigen Variant Prevalence 13 Years After Implementation of a Universal Vaccination Program in China.” Journal of Virology 87: 12196–12206. 24006443 10.1128/JVI.02127-13 PMC3807931

[pbi70270-bib-0004] Chatterjee, M. , S. Gupta , A. Bhar , and S. Das . 2012. “Optimization of an Efficient Protein Extraction Protocol Compatible With Two‐Dimensional Electrophoresis and Mass Spectrometry From Recalcitrant Phenolic Rich Roots of Chickpea (*Cicer arietinum* L.).” International Journal of Proteomics 2012: 536963. 23193474 10.1155/2012/536963 PMC3502011

[pbi70270-bib-0005] Cregg, J. M. , J. F. Tschopp , C. Stillman , et al. 1987. “High–Level Expression and Efficient Assembly of Hepatitis B Surface Antigen in the Methylotrophic Yeast, *Pichia pastoris* .” Nature Biotechnology 5: 479–485.

[pbi70270-bib-0006] Dobrica, M. O. , C. Lazar , and N. Branza‐Nichita . 2020. “N‐Glycosylation and N‐Glycan Processing in HBV Biology and Pathogenesis.” Cells 9: 1404. 32512942 10.3390/cells9061404 PMC7349502

[pbi70270-bib-0007] Dobrica, M. O. , C. Lazar , L. Paruch , et al. 2017. “A Novel Chimeric Hepatitis B Virus S/preS1 Antigen Produced in Mammalian and Plant Cells Elicits Stronger Humoral and Cellular Immune Response Than the Standard Vaccine‐Constituent, S Protein.” Antiviral Research 144: 256–265. 28666757 10.1016/j.antiviral.2017.06.017

[pbi70270-bib-0008] Dorobantu, C. , A. Macovei , C. Lazar , R. A. Dwek , N. Zitzmann , and N. Branza‐Nichita . 2011. “Cholesterol Depletion of Hepatoma Cells Impairs Hepatitis B Virus Envelopment by Altering the Topology of the Large Envelope Protein.” Journal of Virology 85: 13373–13383. 21994451 10.1128/JVI.05423-11 PMC3233160

[pbi70270-bib-0009] Geng, D. , Y. Wang , P. Wang , W. Li , and Y. Sun . 2003. “Stable Expression of Hepatitis B Surface Antigen Gene in *Dunaliella salina* (Chlorophyta).” Journal of Applied Phycology 15: 451–456.

[pbi70270-bib-0010] Gerlich, W. H. 2017. “Do We Need Better Hepatitis B Vaccines?” Indian Journal of Medical Research 145: 414–419. 28862172 10.4103/ijmr.IJMR_1852_16 PMC5663154

[pbi70270-bib-0011] Hammel, A. , L. M. Cucos , I. Caras , et al. 2024. “The Red Alga *Porphyridium* as a Host for Molecular Farming: Efficient Production of Immunologically Active Hepatitis C Virus Glycoprotein.” Proceedings of the National Academy of Sciences, USA 121: e2400145121. 10.1073/pnas.2400145121 PMC11181018 38833465

[pbi70270-bib-0012] Hammel, A. , J. Neupert , and R. Bock . 2024. “Optimized Transgene Expression in the Red Alga *Porphyridium purpureum* and Efficient Recombinant Protein Secretion Into the Culture Medium.” Plant Molecular Biology 114: 18. 38353826 10.1007/s11103-024-01415-2 PMC10866757

[pbi70270-bib-0013] Hempel, F. , J. Lau , A. Klingl , and U. G. Maier . 2011. “Algae as Protein Factories: Expression of a Human Antibody and the Respective Antigen in the Diatom *Phaeodactylum tricornutum* .” PLoS One 6: e28424. 22164289 10.1371/journal.pone.0028424 PMC3229587

[pbi70270-bib-0014] Ho, J. K. , B. Jeevan‐Raj , and H. J. Netter . 2020. “Hepatitis B Virus (HBV) Subviral Particles as Protective Vaccines and Vaccine Platforms.” Viruses 12: 126. 31973017 10.3390/v12020126 PMC7077199

[pbi70270-bib-0015] Huovila, A. P. , A. M. Eder , and S. D. Fuller . 1992. “Hepatitis B Surface Antigen Assembles in a Post‐ER, Pre‐Golgi Compartment.” Journal of Cell Biology 118: 1305–1320. 1522109 10.1083/jcb.118.6.1305 PMC2289601

[pbi70270-bib-0016] Julithe, R. , G. Abou‐Jaoudé , and C. Sureau . 2014. “Modification of the Hepatitis B Virus Envelope Protein Glycosylation Pattern Interferes With Secretion of Viral Particles, Infectivity, and Susceptibility to Neutralizing Antibodies.” Journal of Virology 88: 9049–9059. 24899172 10.1128/JVI.01161-14 PMC4136284

[pbi70270-bib-0017] Lai, M. W. , T. Y. Lin , K. C. Tsao , et al. 2012. “Increased Seroprevalence of HBV DNA With Mutations in the S Gene Among Individuals Greater Than 18 Years Old After Complete Vaccination.” Gastroenterology 143: 400–407. 22580098 10.1053/j.gastro.2012.05.002

[pbi70270-bib-0018] Lazar, C. , D. Durantel , A. Macovei , et al. 2007. “Treatment of Hepatitis B Virus‐Infected Cells With Alpha‐Glucosidase Inhibitors Results in Production of Virions With Altered Molecular Composition and Infectivity.” Antiviral Research 76: 30–37. 17548120 10.1016/j.antiviral.2007.04.004

[pbi70270-bib-0019] Lee, M. C. , H. Y. Yeh , F. J. Jhang , P. T. Lee , Y. K. Lin , and F. H. Nan . 2021. “Enhancing Growth, Phycoerythrin Production, and Pigment Composition in the Red Alga *Colaconema* sp. Through Optimal Environmental Conditions in an Indoor System.” Bioresource Technology 333: 125199. 33930673 10.1016/j.biortech.2021.125199

[pbi70270-bib-0020] Li, W. , H. N. Su , Y. Pu , et al. 2019. “Phycobiliproteins: Molecular Structure, Production, Applications, and Prospects.” Biotechnology Advances 37: 340–353. 30685481 10.1016/j.biotechadv.2019.01.008

[pbi70270-bib-0021] Li, X. , W. Hou , J. Lei , H. Chen , and Q. Wang . 2023. “The Unique Light‐Harvesting System of the Algal Phycobilisome: Structure, Assembly Components, and Functions.” International Journal of Molecular Sciences 24: 9733. 37298688 10.3390/ijms24119733 PMC10254012

[pbi70270-bib-0022] Li, Z. , and R. Bock . 2018. “Replication of Bacterial Plasmids in the Nucleus of the Red Alga *Porphyridium purpureum* .” Nature Communications 9: 3451. 10.1038/s41467-018-05651-1 PMC6110788 30150628

[pbi70270-bib-0023] Pantazica, A. M. , M. O. Dobrica , C. Lazar , et al. 2022. “Efficient Cellular and Humoral Immune Response and Production of Virus‐Neutralizing Antibodies by the Hepatitis B Virus S/preS1^16‐42^ Antigen.” Frontiers in Immunology 13: 941243. 35935966 10.3389/fimmu.2022.941243 PMC9354405

[pbi70270-bib-0024] Pantazica, A. M. , A. van Eerde , M. O. Dobrica , et al. 2023. “The “Humanized” N‐Glycosylation Pathway in CRISPR/Cas9‐Edited *Nicotiana benthamiana* Significantly Enhances the Immunogenicity of a S/preS1 Hepatitis B Virus Antigen and the Virus‐Neutralizing Antibody Response in Vaccinated Mice.” Plant Biotechnology Journal 6: 1176–1190. 10.1111/pbi.14028 PMC10214758 36779605

[pbi70270-bib-0025] Pastor, F. , C. Herrscher , R. Patient , et al. 2019. “Direct Interaction Between the Hepatitis B Virus Core and Envelope Proteins Analyzed in a Cellular Context.” Scientific Reports 9: 16178. 31700077 10.1038/s41598-019-52824-z PMC6838148

[pbi70270-bib-0026] Patient, R. , C. Hourioux , and P. Roingeard . 2009. “Morphogenesis of Hepatitis B Virus and Its Subviral Envelope Particles.” Cellular Microbiology 11: 1561–1570. 19673892 10.1111/j.1462-5822.2009.01363.x PMC2909707

[pbi70270-bib-0027] Prange, R. 2022. “Hepatitis B Virus Movement Through the Hepatocyte: An Update.” Biology of the Cell 114: 325–348. 35984727 10.1111/boc.202200060

[pbi70270-bib-0028] Pulendran, B. , S. Arunachalam , and D. T. O'Hagan . 2021. “Emerging Concepts in the Science of Vaccine Adjuvants.” Nature Reviews Drug Discovery 20: 454–475. 33824489 10.1038/s41573-021-00163-y PMC8023785

[pbi70270-bib-0029] Scaife, M. A. , G. Nguyen , J. Rico , D. Lambert , K. E. Helliwell , and A. G. Smith . 2015. “Establishing *Chlamydomonas reinhardtii* as an Industrial Biotechnology Host.” Plant Journal 82: 532–546. 10.1111/tpj.12781 PMC4515103 25641561

[pbi70270-bib-0030] Schillie, S. , C. Vellozzi , A. Reingold , et al. 2018. “Prevention of Hepatitis B Virus Infection in the United States: Recommendations of the Advisory Committee on Immunization Practices.” MMWR Recommendations and Reports 67, no. 1: 1–31. 10.15585/mmwr.rr6701a1 PMC5837403 29939980

[pbi70270-bib-0031] Seitz, S. , J. Habjanič , A. K. Schütz , and R. Bartenschlager . 2020. “The Hepatitis B Virus Envelope Proteins: Molecular Gymnastics Throughout the Viral Life Cycle.” Annual Review of Virology 7: 263–288. 10.1146/annurev-virology-092818-015508 32600157

[pbi70270-bib-0032] Urban, S. , R. Bartenschlager , R. Kubitz , and F. Zoulim . 2014. “Strategies to Inhibit Entry of HBV and HDV Into Hepatocytes.” Gastroenterology 147: 48–64. 24768844 10.1053/j.gastro.2014.04.030

[pbi70270-bib-0033] Vesikari, T. , A. Finn , P. van Damme , et al. 2021. “Immunogenicity and Safety of a 3‐Antigen Hepatitis B Vaccine vs. a Single‐Antigen Hepatitis B Vaccine: A Phase 3 Randomized Clinical Trial.” JAMA Network Open 4: e2128652. 34636914 10.1001/jamanetworkopen.2021.28652 PMC8511978

[pbi70270-bib-0034] Vesikari, T. , J. M. Langley , V. Popovic , and F. Diaz‐Mitoma . 2023. “PreHevbrio: The First Approved 3‐Antigen Hepatitis B Vaccine.” Expert Review of Vaccines 22: 1041–1054. 37877189 10.1080/14760584.2023.2274482

[pbi70270-bib-0035] Vesikari, T. , J. M. Langley , N. Segall , et al. 2021. “Immunogenicity and Safety of a Tri‐Antigenic Versus a Mono‐Antigenic Hepatitis B Vaccine in Adults (PROTECT): A Randomised, Double‐Blind, Phase 3 Trial.” Lancet Infectious Diseases 21: 1271–1281. 33989539 10.1016/S1473-3099(20)30780-5

[pbi70270-bib-0036] Wang, H. , X. Wang , Z.‐J. Ke , et al. 2015. “Tunicamycin‐Induced Unfolded Protein Response in the Developing Mouse Brain.” Toxicology and Applied Pharmacology 283, no. 3: 157–167. 25620058 10.1016/j.taap.2014.12.019 PMC4361256

[pbi70270-bib-0037] Yoon, D. , J. H. Moon , A. Cho , et al. 2023. “Structure‐Based Insight on the Mechanism of N‐Glycosylation Inhibition by Tunicamycin.” Molecules and Cells 46: 337–344. 37190766 10.14348/molcells.2023.0001 PMC10258461

[pbi70270-bib-0038] Zhao, T. , Y. Cai , Y. Jiang , et al. 2023. “Vaccine Adjuvants: Mechanisms and Platforms.” Signal Transduction and Targeted Therapy 8: 283. 37468460 10.1038/s41392-023-01557-7 PMC10356842

[pbi70270-bib-0039] Zuckerman, A. J. 2000. “Effect of Hepatitis B Virus Mutants on Efficacy of Vaccination.” Lancet 355: 1382–1384. 10791517 10.1016/S0140-6736(00)02132-2

