# Supplementary figures and images for: Red Alga Porphyridium Supports High‐Yield Production of a Functional Chimeric Hepatitis B Surface Antigen With Strong Cellular and Humoral Immunogenicity
Source: Plant Biotechnol J. 2025 Jul 22;23(11):4829–43. doi: 10.1111/pbi.70270 (PMC12576452; doi:10.1111/pbi.70270)

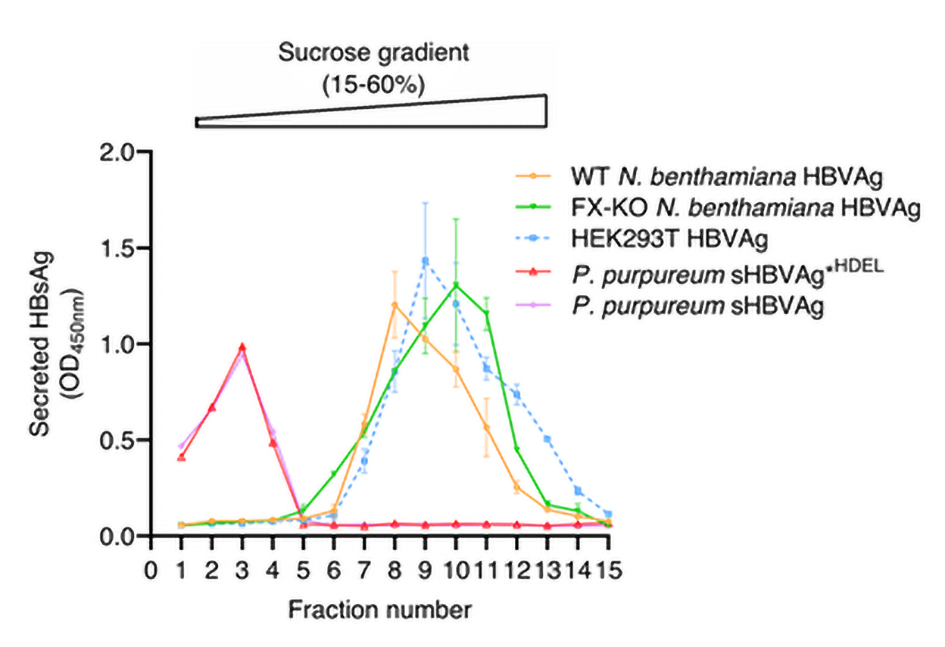

Supplement: Supplementary file 1 — Figure S1. VLP assembly of HA‐tagged HBVAg produced in P. purpureum . Assembly into VLPs of HA‐tagged HBVAg produced in P. purpureum and untagged HBVAg produced in HEK293T and WT or FX‐KO N. benthamiana was verified by sucrose gradient ultracentrifugation. [file PBI-23-4829-s006.tif]

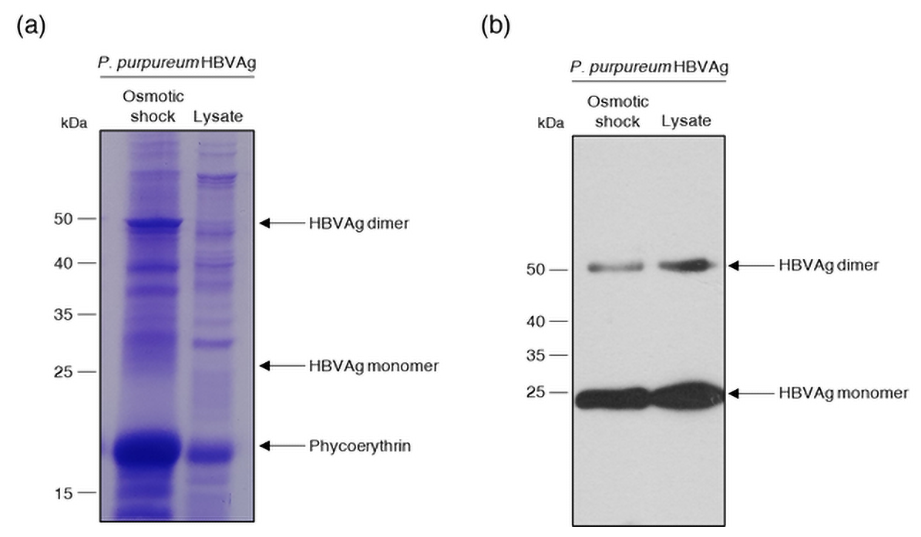

Supplement: Supplementary file 2 — Figure S2. Isolation of HBVAg produced in P. purpureum. Isolation of HBVAg VLPs produced in P. purpureum via osmotic shock followed by cell lysis and analysis of resulting extracts via Coomassie staining and western‐blot. [file PBI-23-4829-s001.tif]

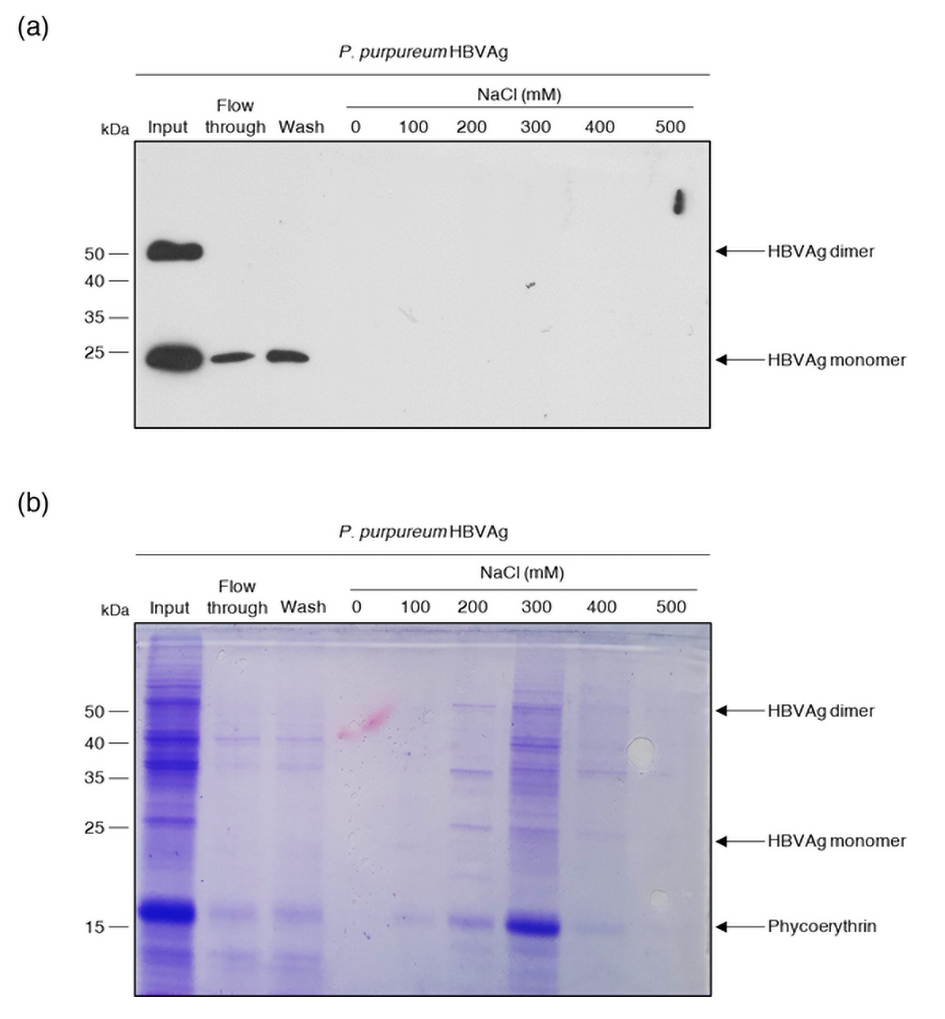

Supplement: Supplementary file 3 — Figure S3. Purification of P. purpureum‐produced HBVAg by ion‐exchange chromatography. Anion exchange chromatography purification of HBVAg produced in P. purpureum and analysis of resulting fractions via Coomassie staining and western‐blot. [file PBI-23-4829-s004.tif]

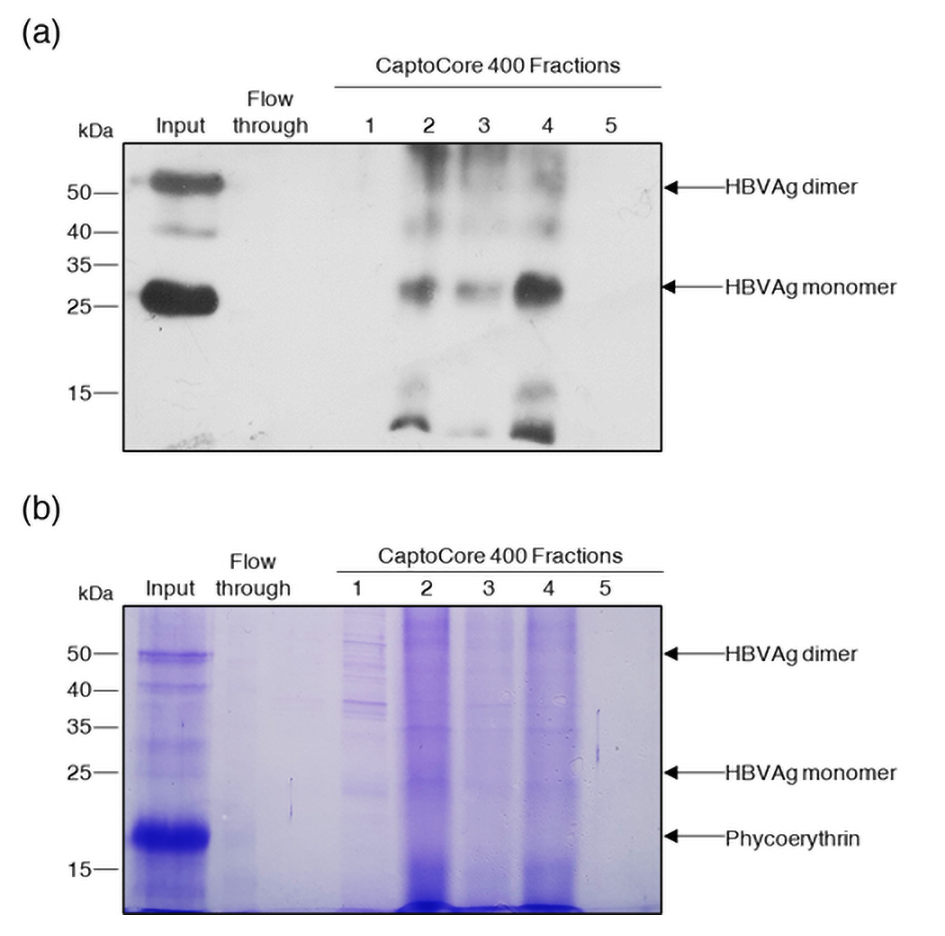

Supplement: Supplementary file 4 — Figure S4. Purification of P. purpureum‐produced HBVAg by gel‐filtration on CaptoCore 400 resin. Size‐exclusion chromatography purification of HBVAg produced in P. purpureum and purified via anion exchange chromatography and analysis of resulting fractions via Coomassie staining and western blot. [file PBI-23-4829-s003.tif]

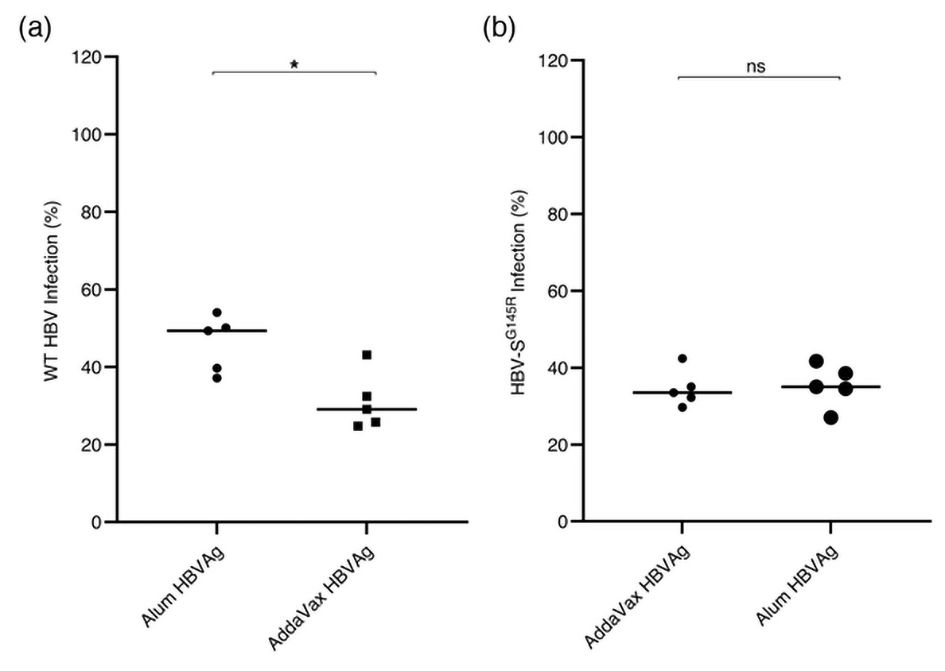

Supplement: Supplementary file 5 — Figure S5. Comparison between adjuvants (Alum vs. AddaVax) and their effects on the neutralisation capacity of sera from mice immunised with P. purpureum ‐expressed HBVAg. WT and VEM HBV neutralisation assays using sera from mice immunised with the P. purpureum ‐produced HBVAg in the presence of either Alum or AddaVax. Infection levels were quantified by ELISA. [file PBI-23-4829-s005.tif]
