# Supplementary material for: Red Alga Porphyridium Supports High‐Yield Production of a Functional Chimeric Hepatitis B Surface Antigen With Strong Cellular and Humoral Immunogenicity
Source: Plant Biotechnol J. 2025 Jul 22;23(11):4829–43. doi: 10.1111/pbi.70270 (PMC12576452; doi:10.1111/pbi.70270)
Supplement: Supplementary file 6 — Table S1. Primer sequences used for vector construction [file PBI-23-4829-s002.docx]

**Table S1. Primer sequences used for vector construction**

| **Construct** | **Primer sequences (5′ - 3′)** |
| --- | --- |
| HBVAg* | **F:** CTCGAGGAGTGCAGGCAACactagtatggagaacatcacgtcgggcttcc  **R:** CTGAAGCCTTGTGAGAGCTAGCGCGACGTCTAGGCGTAATCGGGGACATC |
| sHBVAg* | **F:** CTCGAGGAGTGCAGGCAACactagtATGCGTAAGATGACGCTCACG  **R:** TGAAGCCTTGTGAGAGCTAGCGCGACGTCTAGGCGTAATCGGGGACATC |
| sHBVAg*^-HDEL^ | **F:** CTCGAGGAGTGCAGGCAACactagtATGCGTAAGATGACGCTCACG  **R:** CTGAAGCCTTGTGAGAGCTAGCGCGACGTCTACAGCTCGTCGTGCGAGCC |
| HBVAg | **F:** CTCGAGGAGTGCAGGCAACactagtatggagaacatcacgtcgggcttcc  **R:** CACTCTGAAGCCTTGTGAGAGCTAGCGCGACGTCTAGATGTACACCCACAGGCAGAAGAAG |
